# Unveiling the impact of pipe materials on water hammer in pressure pipelines: an experimental and numerical study

**DOI:** 10.1038/s41598-024-80853-w

**Published:** 2024-12-23

**Authors:** M. Kandil, Tamer A. El-Sayed, A. M. Kamal

**Affiliations:** 1 Department of Mechanical Design, Faculty of Engineering, Helwan University, P.O. Box 11718, Mataria, Helmeiat-Elzaton, Cairo, Egypt; 2 School of Engineering, UH Hosted by Global Academic Foundation, Cairo, Egypt

**Keywords:** Water hammer (WH), Transient Flow, Experimental analysis, Numerical analysis, Dimensionless, Engineering, Mechanical engineering

## Abstract

Water hammer (WH) is a phenomenon characterized by the rapid opening or closing of valves or pumps in pipelines, resulting in a disruptive noise, intense vibrations, and potential damage to pipes, fittings, structures, and even human safety. While WH arresters are commonly employed to mitigate this issue in smaller plumbing systems, alternative solutions are required for larger applications like power plants. Researchers have proposed the utilization of pipe materials with a low modulus of elasticity in areas prone to WH events, as these materials possess the capability to absorb a significant portion of the resulting vibrations. This study focuses on investigating the influence of pressure and strain induced by the WH phenomenon. Experimental data collected from measurements using a specially designed test rig, as well as numerical and dimensionless analyses applied to the same rig, are employed to examine this effect. The experimental and numerical investigations encompass a range of flow rates and pressures. Five pipe materials, namely Galvanized steel, Copper, uPVC, PPr, and GRP, were selected for evaluation. The results indicate a deviation between the numerical and experimental results of WH frequency which can be explained from the pipeline rigidity. A Fast Fourier Transform (FFT) analysis was performed to analyze the frequency components of the pressure transients resulting from water hammer events in the different pipe materials. The results indicate that pipe materials with lower elastic modulus, such as PPr and uPVC, exhibit reduced WH effects compared to materials with higher elastic modulus, such as steel. This research highlights the importance of selecting appropriate pipe materials for pressure pipelines in order to mitigate the dangers associated with WH. The results of the FFT analysis revealed distinct frequency responses for each material, illustrating how their unique viscoelastic properties affect the transient behavior.

**Supplementary Information:**

The online version contains supplementary material available at 10.1038/s41598-024-80853-w.

## Introduction

The fluid flow within pipelines is critical for numerous hydraulic systems, including drinking water supply networks, pipeline networks, industrial power stations, nuclear power plants, and cooling systems. However, alterations in fluid flow conditions resulting from deliberate or accidental events involving hydraulic components can cause sudden pressure variations in the conveyed fluid, leading to a phenomenon known as a WH^1^. A WH arises from the inertial and elastic effects of liquid motion within a pipeline caused by an abrupt valve closure or opening, pump start-up, or shutdown. The severity of the WH is primarily determined by the alteration in the fluid velocity and wave celerity in the pipeline, which are influenced by the pipeline dimensions and material properties. Lower fluid velocities lead to less severe WH. Therefore, understanding the causes and effects of a WH is crucial for ensuring the safe and efficient operation of hydraulic systems. The WH phenomenon as can be caused by sudden valve opening or closing, sudden pump stopping or starting, sudden failure of the mechanics of a hydraulic device, and condensation of steam.

A sudden change in the pressure head of the pipeline that causes a quick pressure wave to propagate through it can cause serious issues, such as noise and vibration or the collapse of the pipeline and its accessories, which may result in fatalities^2–4^. Additionally, there is potential deterioration in water quality due to changes in chlorine content^5^. WH is a commonly observed phenomenon in a range of hydraulic systems such as water distribution networks, hydroelectric power plants, and nuclear power plants. Owing to their widespread occurrence, extensive research has been conducted using a combination of experimental and numerical techniques. Notably, the experimental investigations conducted by Holmboe and Rouleau^6^included two experiments that showed the effect of viscous shear during WH in pipelines. Furthermore, numerous studies have focused on enhancing experimental methodologies and computational modelling^7–10^to advance our understanding of WH. The WH phenomenon has received significant attention from the scientific community, leading to an extensive investigation of its characteristics^11–16^. Theoretical simulations have played a crucial role in studying WH, employing various numerical methods such as the Method of Characteristics (MOC), Finite Differences (FD), Wave Plane (WP), Finite Volumes (FV), and Finite Elements (FE)^11–18^. Among these methods, MOC has emerged as the most popular choice, particularly for complex systems^19^. As a result, it serves as a reference for numerical calculations and forms the basis for computer programs like Flowmeter, Flowmaster, Hammer, Impulse, and Hytran^13,20^.

In simple water pipe systems, numerical investigations have explored the influence of variables such as wave velocity, valve operation time, Reynolds number, and data measurement on transient flow analysis and modeling^21^. The results indicate that the variability of transient flow modeling can be easily influenced by factors such as wave speed, system complexity, and other sources of uncertainty. Researchers have examined several computer programmers to model and replicate WH behavior based on several nontraditional experimental tests^22–24^.

WH waves move through the pipe system as elastic waves and can cause systemic disruptions. Traditional WH theory accurately describes these dynamics for rigid or quasi-rigid structures^25,26^.

Many studies have been studied the Fluid-Structure Interaction (FSI); the scientific literature highlights three primary factors contributing to fluid-structure interaction (FSI) coupling. First, the friction between the pipe wall and the liquid plays a crucial role. Second, the Poisson effect introduces a coupling mechanism that links the changes in the liquid pressure to the longitudinal strains within the pipe. Third, the junction coupling (JC) effect occurs at pipe bends, ends, valves, flow throttling components, and other locations where a significant FSI can occur. The JC effect becomes particularly pronounced when the pipeline can move as a unit, such as in the presence of an elastic pipe support. Consequently, owing to the motion of the pipe, energy can be transferred from the liquid to the structure primarily at the supports, resulting in dissipation. This phenomenon results in pressure fluctuations, additional stresses, and deformations in the pipe system. It is important to consider all three factors when modeling the FSI coupling in pipes to accurately predict the behavior of the system^27–30^.

Fluid-structure interaction (FSI) can arise in a flexibly supported pipe–fluid system owing to the WH phenomenon, and researchers have explored this phenomenon using hybrid models^17–19,31^.

In the FSI equation system, the liquid equations should be adjusted properly to consider the longitudinal pipe motion. For the pipe motion, two additional partial differential equations were created, resulting in the four-equation WH-FSI model^32^ .

Many studies have been studied the viscoelasticity and material effects such as^33–36^; Kandil et al.^37^ investigated the influence of pipe materials on transient waves and demonstrated that low elastic modulus materials can enhance the amplitude of WH pressure. Soares et al.^38^explored a model for analyzing the transient behavior in polyvinyl chloride (PVC) pipes through numerous experiments and showed a better alignment between experimental and numerical results with the proposed model. It is crucial to compare the results of numerical models of WH behavior with the results of experimental studies to ascertain whether they can be used for WH analysis and prediction. Triki^39^examined the transient pressure during WH events in metallic pipes protected by composite materials and recommended this technique to enhance the resistance of steel piping systems. In this study, an inline design technique was employed to analyze WH behavior in an existing steel piping system. The approach involves replacing a brief section of the steel pipeline with a different type of plastic pipe wall material, such as high-density polyethylene (HDPE) or low-density polyethylene (LDPE). Keramat et al^40^. proposed a novel numerical simulation method for simulating WH in a plastic pipeline. During the development of the model, the Poisson’s ratio of the plastic pipeline was considered. Laiq et al.^29^ conducted a comparative study of various pipeline materials, namely high-density polyethylene (HDPE), glass-fiber reinforced plastic (GRP), and ductile iron (DI), with respect to their cost efficiency for mitigating the WH phenomenon in pipelines transporting treated sewage effluent (TSE). Through hydraulic analyses, the authors found that GRP pipelines demonstrated superior attenuation of WH pressure waves compared to HDPE and DI pipelines, likely owing to the higher elastic modulus and strength of the GRP. Hence, among the materials considered, GRP pipelines were deemed to be the most effective and economical solution for controlling WH in TSE pipelines. In earlier numerical studies, the focus was primarily on employing a steady friction model, in which the friction factor obtained from the initial condition was assumed to remain constant throughout the transient simulation. However, experimental studies have shown that significant irregularities exist in the attenuation and phase of the pressure oscillations. This has led researchers to incorporate unsteady or transient friction into the momentum equation. See ref.^41–43^. The modeling of unsteady friction has received considerable attention in academic literature, with extensive discussions and investigations conducted on this topic.

The majority of commercial WH analysis software was created with the friction factor (*f *) of Darcy Weisbach as a constant (steady state friction factor)^41^. Adamkowski et al.^44^experimentally investigated the damping effect on pressure waves during a WH, and found that the classic WH theory fails to accurately predict the damping effect. To provide a more detailed description of pipe motion, they developed an extended model comprising fourteen equations, including ten for rotating and transverse pipe vibrations^45,46^. Duan et al.^47^ employed a quasi-2D numerical approach to investigate the influence of unsteady friction in viscoelastic pipes. They concluded that the basic equations for transient motion fail to capture damping behavior.

Hachem and Schleiss^48^ conducted an in-depth analysis of a novel physical experimentation approach that involved the evaluation of pressure and vibration recordings obtained at both ends of a multireach steel test pipe. The main objective of their study was to develop and evaluate a new signal-processing method specifically designed to detect the presence of a weak reach caused by WH waves within the test pipe. Gong et al.^49^ replaced a short piece of an existing hard piping system mode from mild steel with a branching elastic pipeline to study the WH impact and found that this technique can improve the existing steel piping systems against WH effects. Larson et al.^50^ experimentally examined the WH effect in domestic water and sanitary drainage piping networks. They presented the results of the head and strain measurements during WH events for three different piping systems composed of various materials. The study concluded that the linear elasticity theory can be utilized to analyze pipe behavior.

Kawaguchi et al.^51^ employed fracture analysis techniques to study the transient behavior of three distinct types of composite materials, revealing that the resistance to WH is affected by the specimen shape. Choon et al.^52^Laboratory tests on two materials, mild steel (MS) and polyvinyl chloride (PVC), used to investigate the effect of WH, have concluded that materials with a higher modulus of elasticity exhibit good performance with transient effects. This contradicts the findings of this research and the results found in most of the research published in this regard^29,39,53^. Covas et al.^54^ created a new mathematical model based on the (MOC) to investigate the effect of a WH on a viscoelasticity pipe wall based on experimental and numerical findings. Pezzinga et al.^55^ studied transients in pressurised polymeric pipelines was examined using a two-dimensional (2D) Kelvin-Voigt for the viscoelastic pipeline model using a microgenetic method based on wave velocity. The proposed model was validated by comparing the numerical and experimental results of the wave velocity during a WH. Sun et al.^56^ analyzed the response of composite pipes under the influence of WH events and investigated the velocities of the resulting WH waves. Apollonio et al.^57^ used the viscoelasticity model to obtain the transient stresses using the creep test for high-density polyethylene piping. Kodura et al.^58^ conducted experimental investigations to examine the impact of valve closure on WH events in mild steel and plastic pipes. Gustafson et al.^52^ investigated the evolution of transient fatigue in composite (GRE) pipes. Kumar et al.^59^ demonstrated that wave velocity plays a crucial role in determining the amplitude of the WH pressure. Mery et al.^60^ experimentally and numerically studied the effect of air vessels and HDPE forward configuration techniques on induced WH pressure waves. Volkov et al.^61^ studied experimentally and numerically investigate condensation-induced WH (CIWH) phenomena in thermal systems. The study aims to analyze the behavior of CIWH during the filling of a horizontal pipe with subcooled water, focusing on the interactions between steam and subcooled water that can lead to significant pressure waves and potential safety risks.

This study aims to investigate how different pipe materials affect WH in pressure pipelines, and whether there are significant variations in the intensity and frequency of WH among pipes constructed from distinct materials. The objective of this study was to develop a numerical model, validate its accuracy through physical tests, and evaluate its reliability in predicting WH in pipelines with different materials. The research methodology utilized both experimental and numerical techniques to investigate the effects of pipe materials on a WH. An experimental setup with pressure sensors and strain gauges installed at different locations along the pipeline was used to measure the pressure transients generated during the WH events. Numerical simulations using the method of characteristics (MOC) were conducted to model WH events in a pipeline system. This study employs a comprehensive approach that integrates both experimental and numerical methods to investigate the impact of pipe materials on WH in pressure pipelines, and to provide recommendations for selecting suitable pipe materials to mitigate the effects of WH. Section 2 describes the experimental setup, including the test rig, instrumentation, and data-acquisition system. The strain measurements for experimental validation are also discussed. Section 3 presents and analyzes the experimental results obtained by testing different pipe materials under various flow conditions using pressure transducers and strain gauges. Section 4 outlines the numerical model used to simulate the WH in pressure pipelines, including the mathematical equations and dimensionless parameters, investigates the application of FFT analysis to WH phenomena in pipelines constructed from five distinct materials. The FFT technique enables the identification of dominant frequencies and their corresponding amplitudes, providing valuable insights into the dynamic behavior of the pipeline systems. The results reveal that each pipe material exhibits a unique frequency spectrum, reflective of its inherent characteristics. Section 5 presents and compares the numerical simulation results with experimental data to validate the numerical model. Finally, Sect. 6 summarizes the key findings.

## Experimental works

A specialized test rig was designed and constructed at the Mechanics of the Materials Laboratory of the Faculty of Engineering, Materia, Helwan University, Cairo, Egypt. The primary objective of this investigation was to study the phenomenon of WH by monitoring pressure fluctuations over time in five distinct pipelines, each constructed from different materials.

**Fig. 1:**
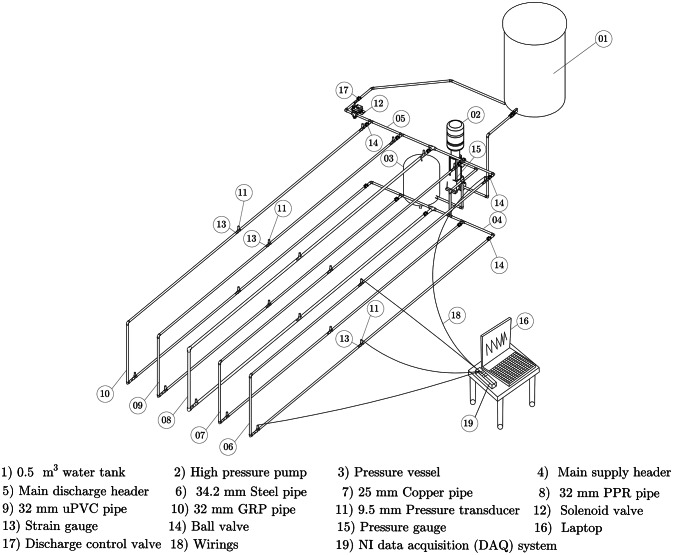
Three-dimensional schematic drawing of the WH test rig featuring four different materials used in pipeline construction.

**Fig. 2:**
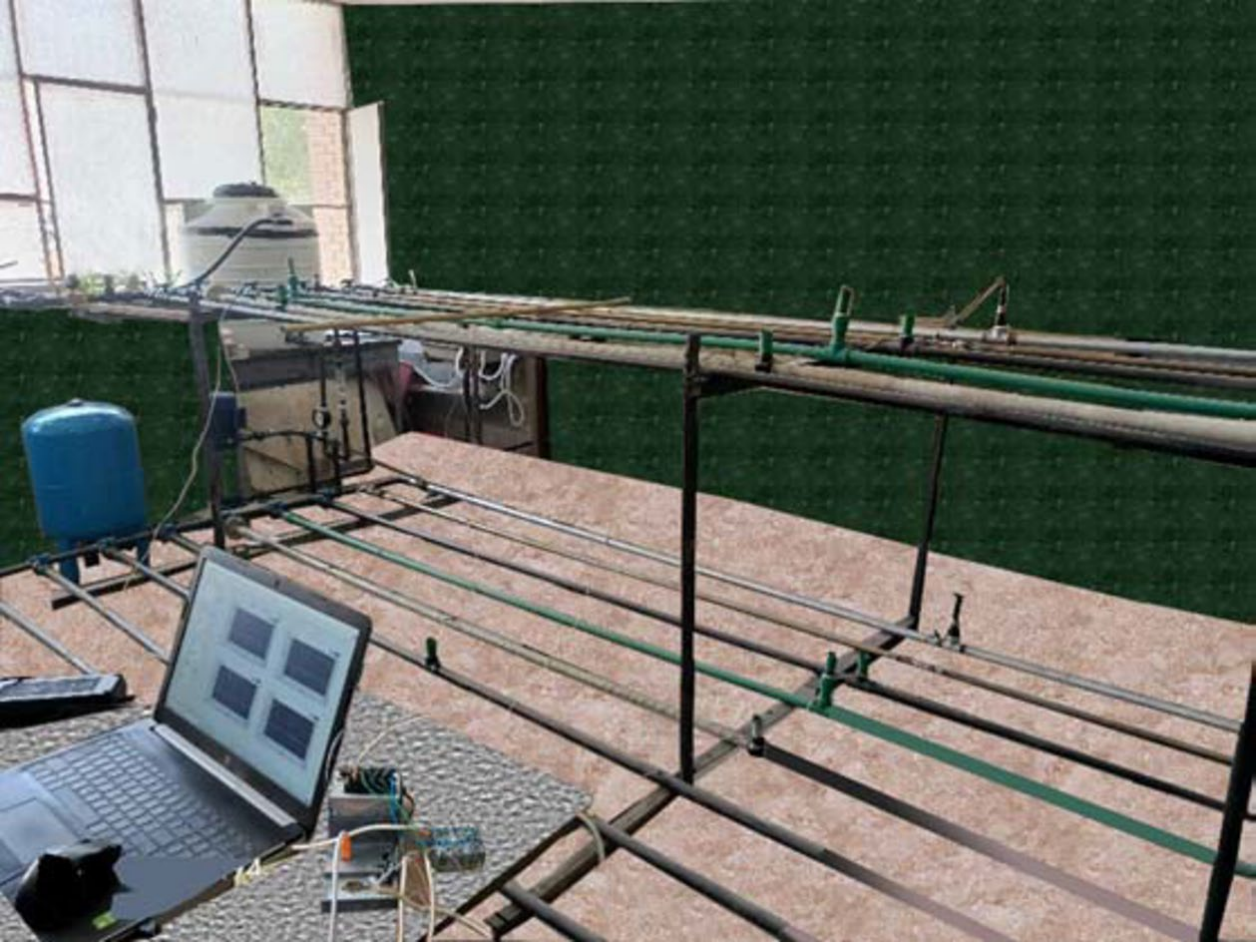
A real photograph of the WH test rig, displaying the pipelines made from five different materials.

Figures 1 and 2:  A real photograph of the WH test rig, displaying the pipelines made from five different materials. depict the schematic layout and an actual photograph of the experimental test rig, respectively.  The system utilized a closed-loop configuration, which incorporated an upstream 0.5 m^3^ polyethylene (PE) water tank.  The system also included a multi-stage high-pressure pump produced by the USA Pedrollo company with a head of 110 bar and a flow rate of 100 L/min, a flow meter with a capacity of 120 L/min, an electrical wiring, a data acquisition (DAQ) system from National Instruments (NI), a laptop running the LabVIEW program, and a pressure gauge with a range of 27 bar. During pump shutoff, a 50-liter pressure vessel was used to maintain a constant pressure on the pipelines. The pressure vessel was filled with compressed air at a pressure of 2 bar in accordance with the manufacturer’s recommendations.

To trigger the WH events within the pipelines, an electrical solenoid valve with a diameter of 25 mm and a closing time of 0.02 s was placed downstream of each pipeline. Additionally, a discharge control valve was installed near the water tank to regulate liquid flow within the pipelines.

1 $$\:\:\:\:\:\:\:\:{E}_{11}={E}_{f}{V}_{f}+{E}_{m}{V}_{m}$$

2 $$\:\:\:\:\:{v}_{12}={v}_{f}{V}_{f}+{v}_{m}{V}_{m}$$

3 $$\:{V}_{m}=\:{1-V}_{f}$$

4 $$\:\frac{1}{{E}_{22}}=\frac{{V}_{f}}{{E}_{f}}+\frac{{V}_{m}}{{E}_{m}}$$

5 $$\:\:\:\:\:{v}_{21}=\frac{{E}_{22}}{{E}_{11}}{v}_{12}$$

6 $$\:\frac{1}{{G}_{12}}=\frac{{V}_{f}}{{G}_{f}}+\frac{{V}_{m}}{{G}_{m}}$$

where *E*11 is the modulus of elasticity of the lamina in the fiber direction, *E*22 is the modulus of elasticity of the lamina transverse to the fiber direction, *Ef* is the fiber modulus of elasticity, *Em* is the matrix modulus of elasticity, *Vf* is the fiber volume fraction, *Vm* is the matrix volume fraction, *v*12 is the major Poisson ratio of the lamina, *v*21 is the minor Poisson ratio of the lamina, *G*12 is the in-plane shear modulus of the lamina, *Gf* is the fiber shear modulus, and *Gm* is the matrix shear modulus (Table 1).

**Table 1:** Physical and mechanical properties of the pipes used in the present experiment^60^.

Pipe Material	Outer Diameter	Inner Diameter	Density	Poisson’s Ratio	Modules of Elasticity $\:\left(\varvec{E}\right)$	Yield Strength $\:\left({\varvec{\sigma\:}}_{\varvec{y}}\right)$
	(mm)	(mm)	(kg/m^3^)		(GPa)	(MPa)
galv St	34.2	27.8	7850	0.30	210	400
Cu	25.0	23.2	8900	0.36	110	105
uPVC	32.0	27.2	1430	0.40	3.38	42.22
PPR	32.0	21.2	900	0.36	0.900	29
GRP	29.8	22.5	1281	-	-	-

**Fig. 3:**
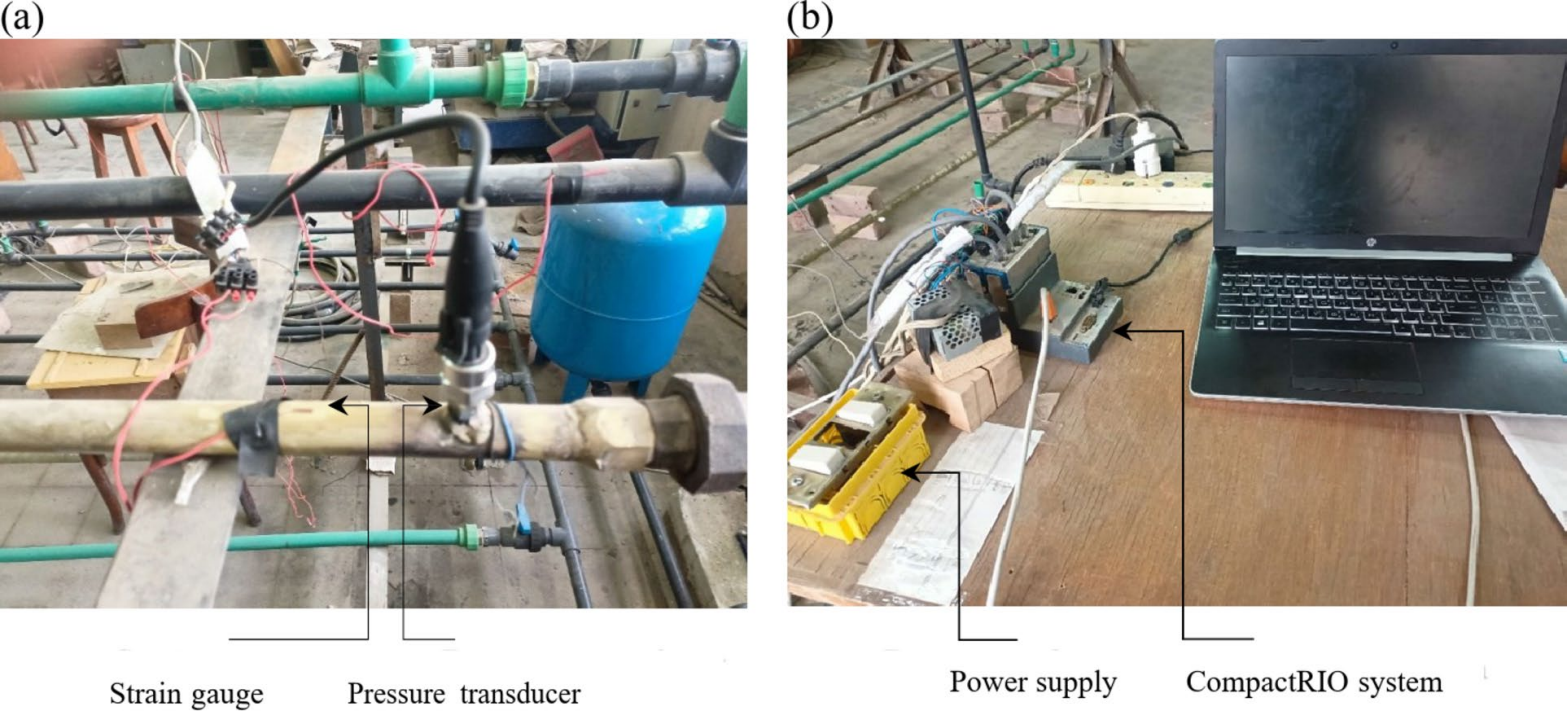
(**a**) Pressure transducers and strain gauges installation on the test pipelines. (**b**) Power supply and NI data acquisition (DAQ) system.

 To investigate the WH phenomenon in piping systems, pressure transducers were placed at critical locations along the piping system. These locations, including areas near valves, bends, and supports, were selected based on their susceptibility to high stress during WH events. Consequently, the final positions of the pressure transducers were determined to be 3, 5.5, 9, and 12 m from the main supply header. Figure 3(a) illustrates the installation of pressure transducers on the tested pipelines. In the investigation of the structural behavior and integrity of a piping WH test rig, the measurement of strain is of paramount importance. To this end, strain gauges were carefully positioned in close proximity to the pressure transducers, allowing for precise measurement of the corresponding axial strains resulting from the WH pressure. The placement of the strain gauges is shown in Fig. 3(a), which depicts their installation on pipelines subjected to testing.

The experimental conditions were set to 5.5 bar at 60 L/min, 6.9 bar at 50 L/min, and 8.3 bar at 40 L/min to create a gradual pressure variation, starting from an average range of 5.5 bar and increasing towards a higher pressure of 8.3 bar. These specific values were chosen to simulate real conditions for domestic water pipeline applications. Figure 3(b), shows the data-acquisition setup utilized in the experimental investigation. During testing, a 5 V DC power supply was utilized to power the pressure transducers, and the initial raw signals obtained from these transducers were in the range of 0 to 5 V. To facilitate data acquisition, we employed National Instruments (NI) cards: NI 9215 for pressure readings and NI 9237 for strain readings.

Both cards were connected to a laptop via NI compactRIO (NI Crio-9063), and data acquisition was carried out through a program that was developed using National Instruments LabVIEW software. To convert voltage measurements obtained from pressure transducers into pressure units (bars), we referred to calibration numbers specified in pressure transducer manufacturer’s data sheet and applied them within LabVIEW software. This allowed us to obtain accurate pressure readings during testing.

### Experimental validation of strain readings

**Fig. 4:**
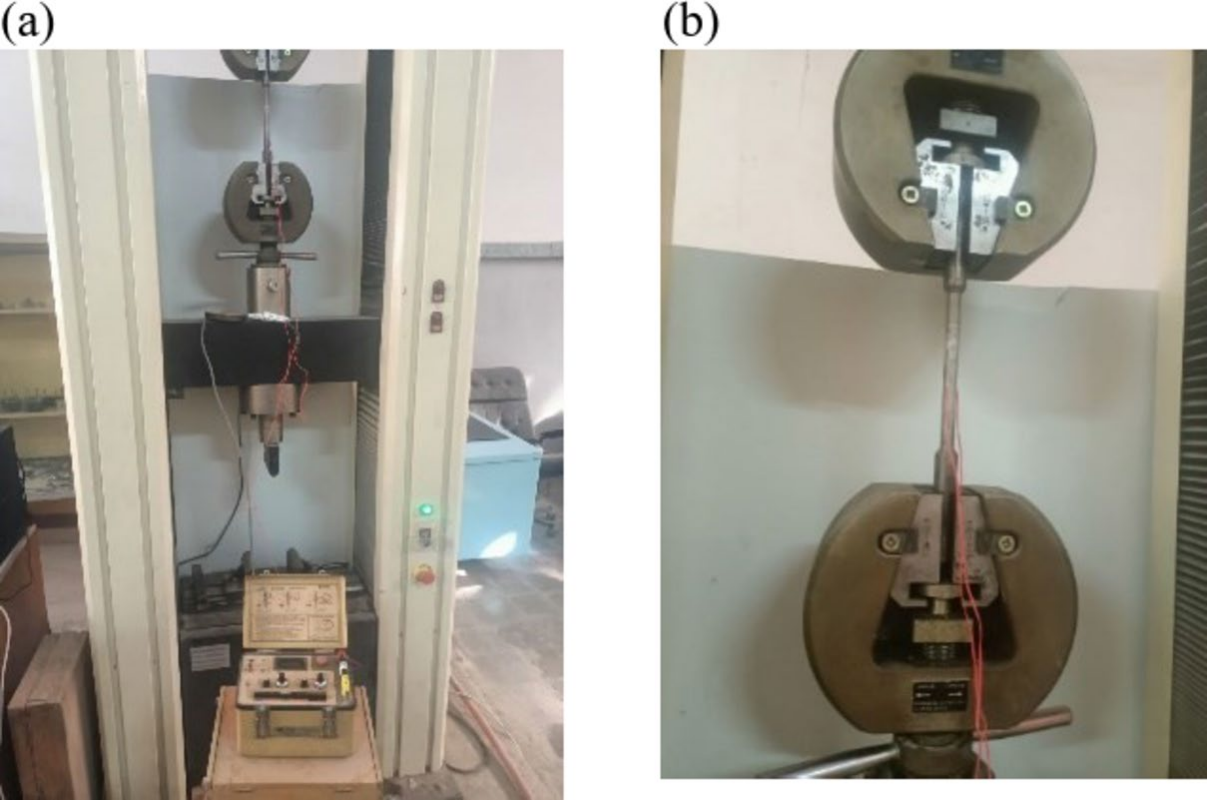
(**a**) Validation of strain readings using tensile test machine and strain meter. (**b**) Steel specimen strain gauge connection.

**Fig. 5:**
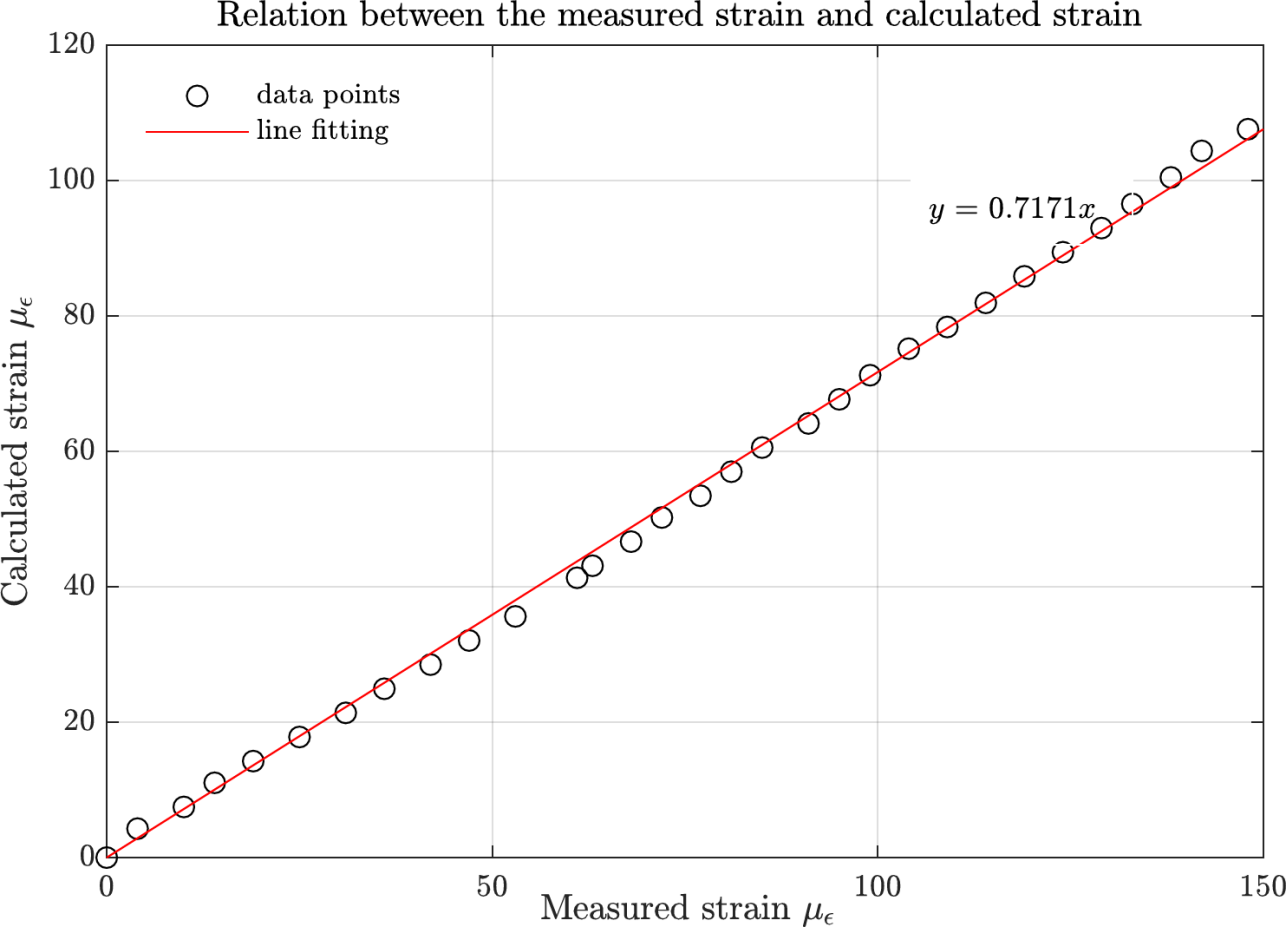
Relation between the measured strain using ni-9237 card and analytical values of strain.

The strain readings measured using NI 9237 card underwent validation through a tensile test conducted on a steel specimen, in accordance with ASTM E8 / E8M, as depicted in Fig. 4. The P-3500 strain indicator readings served to ascertain the modulus of elasticity for the steel specimen under loads up to 12 kN. The NI 9237 card readings facilitated the establishment of a relationship between them and the calculated strains ($\:\epsilon\:=\frac{\sigma\:}{E}$) under loads up to 3 kN. This utilized the modulus of elasticity determined by the strain meter readings, as shown in Fig. 5. The findings indicate that a correction factor of approximately 0.7 is necessary for the NI card strain readings. This correction was applied to the strain measurements of the test rig pipelines, which were predominantly at low strain values.

In the process of validating strain calculations for thick pipes, the equations employed are predicated on elasticity theory, with the assumption that longitudinal

deformation is symmetric about the axis. For a cylinder characterized by a thick wall, an inner radius of (Ri), and an outer radius of (*Ro*), and subjected to internal pressure (*Pi*), the material adheres to Hooke’s law within the elastic range. This permits the utilization of Lame’s equations to compute the hoop stress (*σθ* ), longitudinal stresses (*σl*), and radial stress (*σr* ) across the thickness of the cylinder’s wall. Both *σθ* and *σr* are dependent on the radial position (*r*).

7 $$\:{\sigma\:}_{\theta\:}=\frac{{({R}_{i}}^{2} {P}_{i})}{({{R}_{o}}^{2}-{{R}_{i}}^{2})}\left(1+\frac{{{R}_{o}}^{2}}{{r}^{2}}\right)$$

8 $$\:{\sigma\:}_{r}=\frac{{({R}_{i}}^{2}{P}_{i})}{({{R}_{o}}^{2}-{{R}_{i}}^{2})}\left(1-\frac{{{R}_{o}}^{2}}{{r}^{2}}\right)$$

9 $$\:{\sigma\:}_{l}=\:\frac{{{R}_{i}}^{2}{P}_{i}}{{{R}_{o}}^{2}-{{R}_{i}}^{2}}\:$$

The strain for the three directions can be obtained as

10 $$\:{\epsilon\:}_{\theta\:}=\frac{1}{E}\left[{\sigma\:}_{\theta\:}-v({\sigma\:}_{r}+{\sigma\:}_{l})\right]$$

11 $$\:{\epsilon\:}_{r}=\frac{1}{E}\left[{\sigma\:}_{r}-v({\sigma\:}_{\theta\:}+{\sigma\:}_{l})\right]$$

12 $$\:{\epsilon\:}_{l}=\frac{1}{E}\left[{\sigma\:}_{l}-v({\sigma\:}_{\theta\:}+{\sigma\:}_{r})\right]$$

**Fig. 6:**
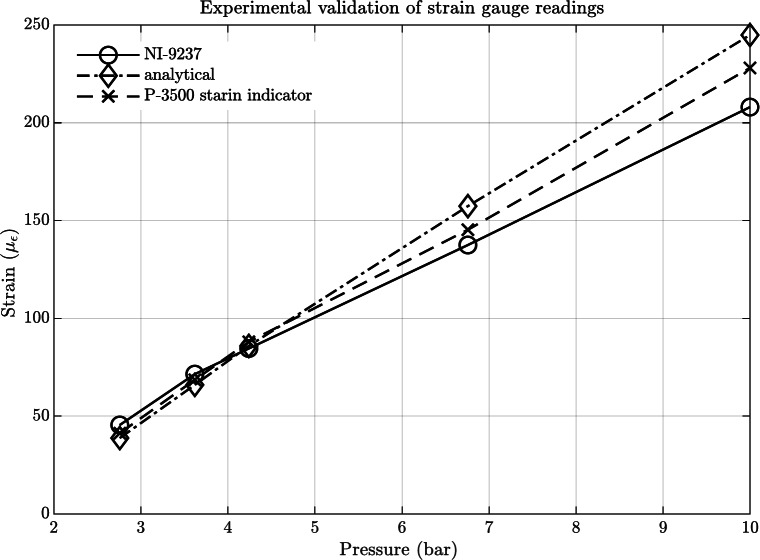
The Relationship Between NI-9237 Card, P-3500 Strain Indicator Readings, and Analytical Values.

Figure 6 depicts the experimental validation results obtained for the strain gauge. A total of five tests were conducted, each at different pressures: 2.7, 3.6, 4.2, 6.7, and 10 bar, with corresponding flow rates. For each pressure condition, the longitudinal strain was measured utilizing both the strain meter and the NI card, following the application of the correction factor within the LabVIEW program. Additionally, the strains can be calculated using Eqs. 10 to 12. As observed in Fig. 6, the values obtained from the three methods exhibit close agreement up to a pressure of 4.2 bar, demonstrating a high level of similarity. However, a slight deviation becomes apparent at higher pressures.

## Experimental results

**Fig. 7:**
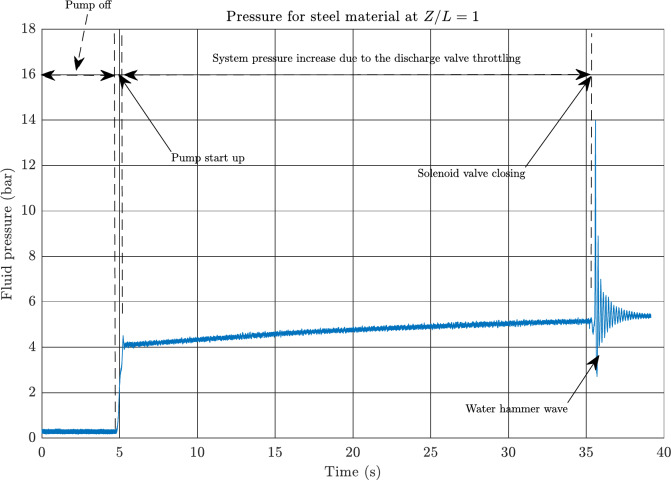
Pressure measurement data for the steel pipeline at pump flow rates of 60 L/min and 5.5 bar pressure at *Z/L* = 1.

The pressure and corresponding axial strain were measured simultaneously for each pipeline. To ensure accurate strain measurements, the LabVIEW software measurement program was initiated before﻿ the﻿ pump turning on, as depicted in Fig. 7. The pump was slowly activated until the system reached a normal pressure of approximately 4.1 bar, as indicated by the pressure gauge. The pressure was then further increased to the required level for each test case by throttling the discharge ball valve. Once the necessary pressure was attained, the solenoid valve was closed to generate a WH effect in the pipeline.

The process of measuring strain is well known for its high level of accuracy; however, it is also considered quite challenging. This is because a careful preparation of the piping surfaces is required before conducting the strain gauge testing, as well as ensuring proper connections to the strain gauges and accounting for other factors. Consequently, the readings obtained often contain a considerable amount of noise. To mitigate this problem, the experimental strain data were filtered using discrete Meyer wavelet filters.

### WH *dimensionless analysis*

In this study, the authors employed the dimensionless parameter of the ratio between WH pressure (P) and the modulus of elasticity (E) of the pipe material to elucidate the effect of the pipe material on the WH phenomenon. By altering the non-dimensional factors, specifically the ratio of WH pressure to modulus of elasticity (P/E), the impact on the speed of WH waves can be explained as follows:

The speed of WH waves is directly proportional to the square root of the ratio between the modulus of elasticity of the pipe material (E) and the fluid density (ρ), as shown in the following equation:

$$c=\sqrt{E/\rho}$$

Where:

*c* is the speed of the WH wave.

*E* is the modulus of elasticity of the pipe material.

*ρ* is the fluid density.

By altering the non-dimensional parameter *P/E*, the authors investigate how changes in the pipe material’s modulus of elasticity affect the propagation of WH waves. This approach allows the authors to elucidate the relationship between the pipe material properties and the WH phenomenon.

The fundamental equation in WH theory, as described by Eq. (13), establishes a relationship between pressure changes (∆*P* ) and velocity changes (∆*V* ). In this equation, *ρ* represents the fluid density and *a*denotes the speed of the WH wave^66^.

13 $$\:\varDelta\:P=\:\rho\:a\:\varDelta\:V$$

Instantaneous valve closure, resulting in WH, is defined as the closure of the valve at a time shorter than the time required for the wave to propagate to and back from the upstream end. This time, known as the wave travel time (*t*_*wh*_), is defined by using Eq. (14), where *twh* represents the wave travel time and *L* denotes the pipe length.

14 $$\:{t}_{wh}=\:\raisebox{1ex}{$2L$}\!\left/\:\!\raisebox{-1ex}{$a$}\right.$$

Referring to Eqs. (13, 14), the speed of the WH wave can be deduced from the rise in pressure and the changes in velocity caused by WH. Furthermore, the speed of the WH wave depends on the pipe length and the valve closing time. Considering Eq. (15) for wave speed, the modulus of elasticity (*E*) plays a crucial role in assessing.

15 $$\:{a}^{2}=\left(\frac{{K}_{bm}}{{\rho\:}_{f}}\right){\left(1+\frac{{K}_{bm}2{R}_{i}}{Eh}\right)}^{-1}$$

where, *Kbm* denotes the fluid bulk modulus in (Pa), *E* represents the modulus of elasticity of the piping in (Pa), and *ν* is the Poisson ratio. The inner radius of the piping is given by *Ri* (m), and the fluid density is represented by *ρ*_*f*_ (kg/m3).

The wave speed is directly proportional to the pipe modulus of elasticity. As the modulus of elasticity of the pipe material increases, the denominator of the equation becomes larger, resulting in a smaller value for the term $\:\left(1+\frac{{K}_{bm}2{R}_{i}}{Eh}\right)$Since the wave speed is inversely proportional to this term, a larger modulus of elasticity leads to a higher WH wave speed.

Table 2 provides information on the specific values of the ratio of pipe wall thickness to inner diameter for the different materials studied.

**Table 2:** The specific values of the ratio of pipe wall thickness to inner diameter for the different materials studied.

Pipe Material	galv St	Cu	uPVC		PPR	GRP E
*h/d*	0.115	0.038	0.088	0.25		0.15

The variation in the ratio of pipe wall thickness to pipe diameter (*h/d*) among different materials, as presented in Table 2, significantly influences the occurrence of WH. This nondimensional factor, *h/d*, directly affects the structural characteristics of the pipe.

The *h/d* ratio plays a crucial role in determining the stiffness of the pipe, thereby influencing the speed of the WH wave. Referring to Eq. (15), it can be observed that a higher *h/d* ratio generally, enhances the rigidity of the pipe, resulting in a higher wave speed. Conversely, a lower *h/d* ratio leads to a lower wave speed.

**Fig. 8:**
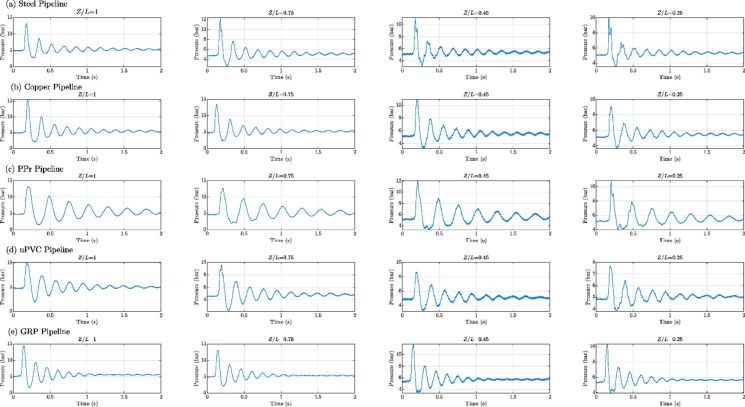
Experimental results of transient pressures measured at four specific locations for a pump flow rate of 60 L/min and a pressure of 5.5 bar: (**a**) Galvanized Steel, (**b**) Copper, (**c**) Polypropylene Random Copolymer (PPR), (**d**) Unplasticized Polyvinyl Chloride (uPVC), and (**e**) Glass Reinforced Plastic (GRP) pipelines.

**Fig. 9:**
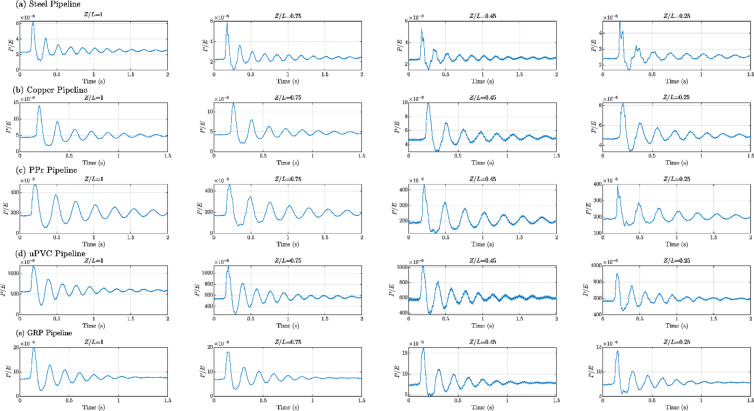
Experimental results depicting transient pressures ratio (*P/E*) measured at four specific locations for a pump flow rate of 60 L/min and a pressure of 5.5 bar: (**a**) Galvanized Steel, (**b**) Copper, (**c**) Polypropylene Random Copolymer (PPR), (**d**) Unplasticized Polyvinyl Chloride (uPVC), and (**e**) Glass Reinforced Plastic (GRP) pipelines.

**Fig. 10:**
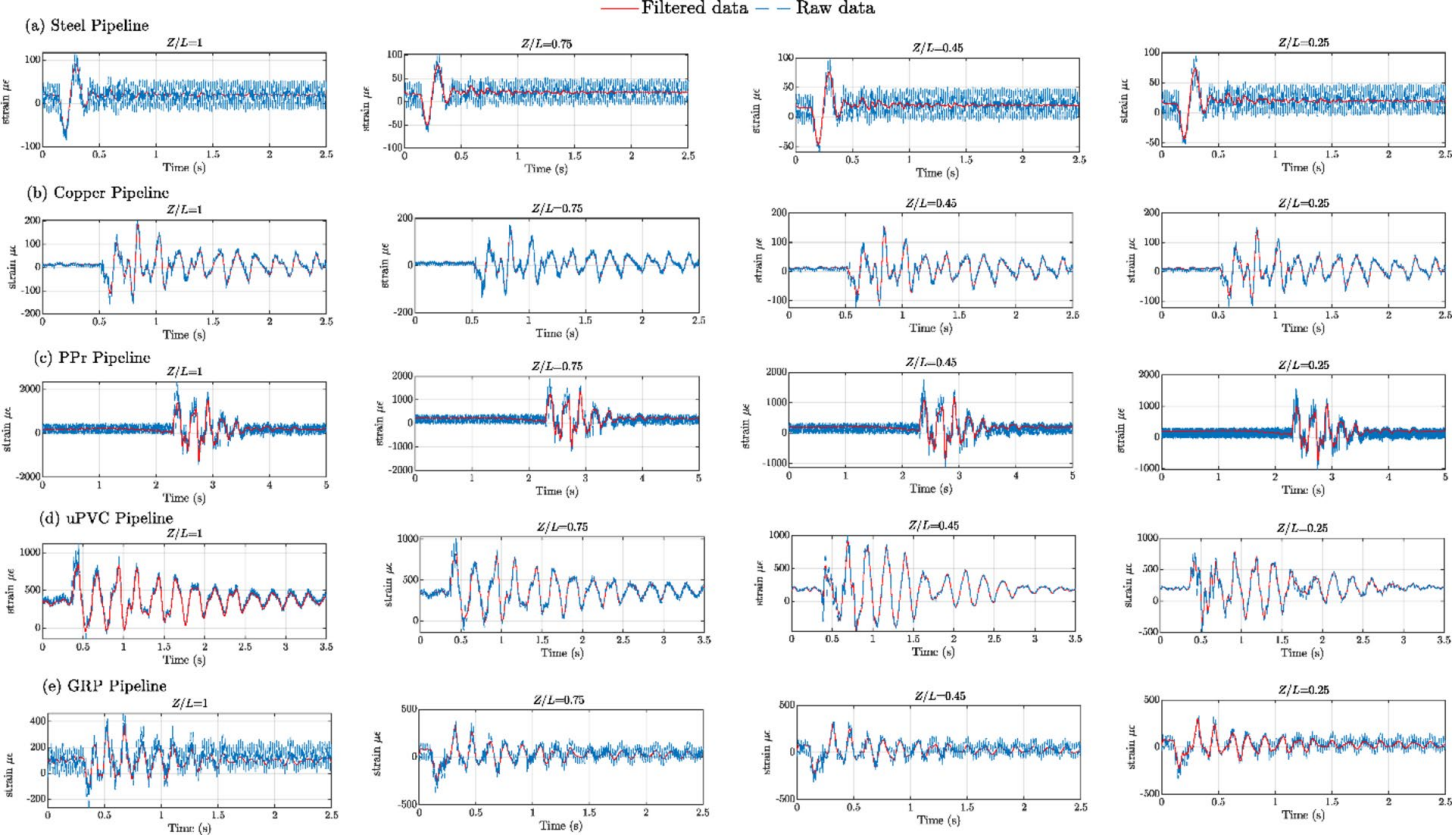
Experimental results displaying corresponding axial strain measurements (in microstrain, ${\mu}\epsilon$) at four distinct locations for a pump flow rate of 60 L/min and a pressure of 5.5 bar: (**a**) Galvanized Steel, (**b**) Copper, (**c**) Polypropylene Random Copolymer (PPR), (**d**) Unplasticized Polyvinyl Chloride (uPVC), and (**e**) Glass Reinforced Plastic (GRP) pipelines.

**Fig. 11:**
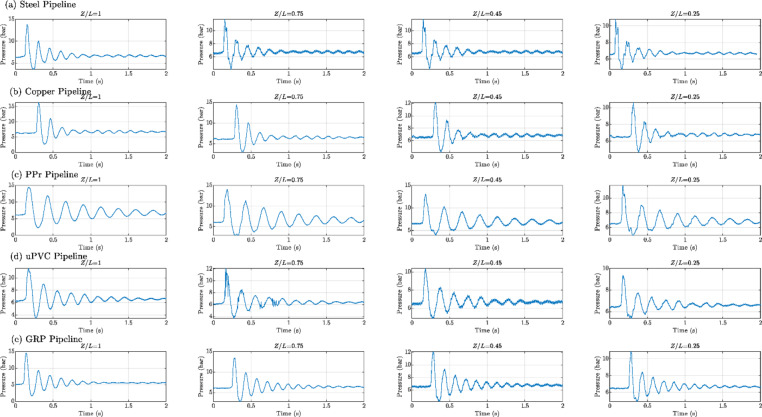
Experimental results illustrating transient pressures at four specified locations for a pump flow rate of 50 L/min and a pressure of 6.9 bar: (**a**) Galvanized Steel, (**b**) Copper, (**c**) Polypropylene Random Copolymer (PPR), (**d**) Unplasticized Polyvinyl Chloride (uPVC), and (**e**) Glass Reinforced Plastic (GRP) pipe.

**Fig. 12:**
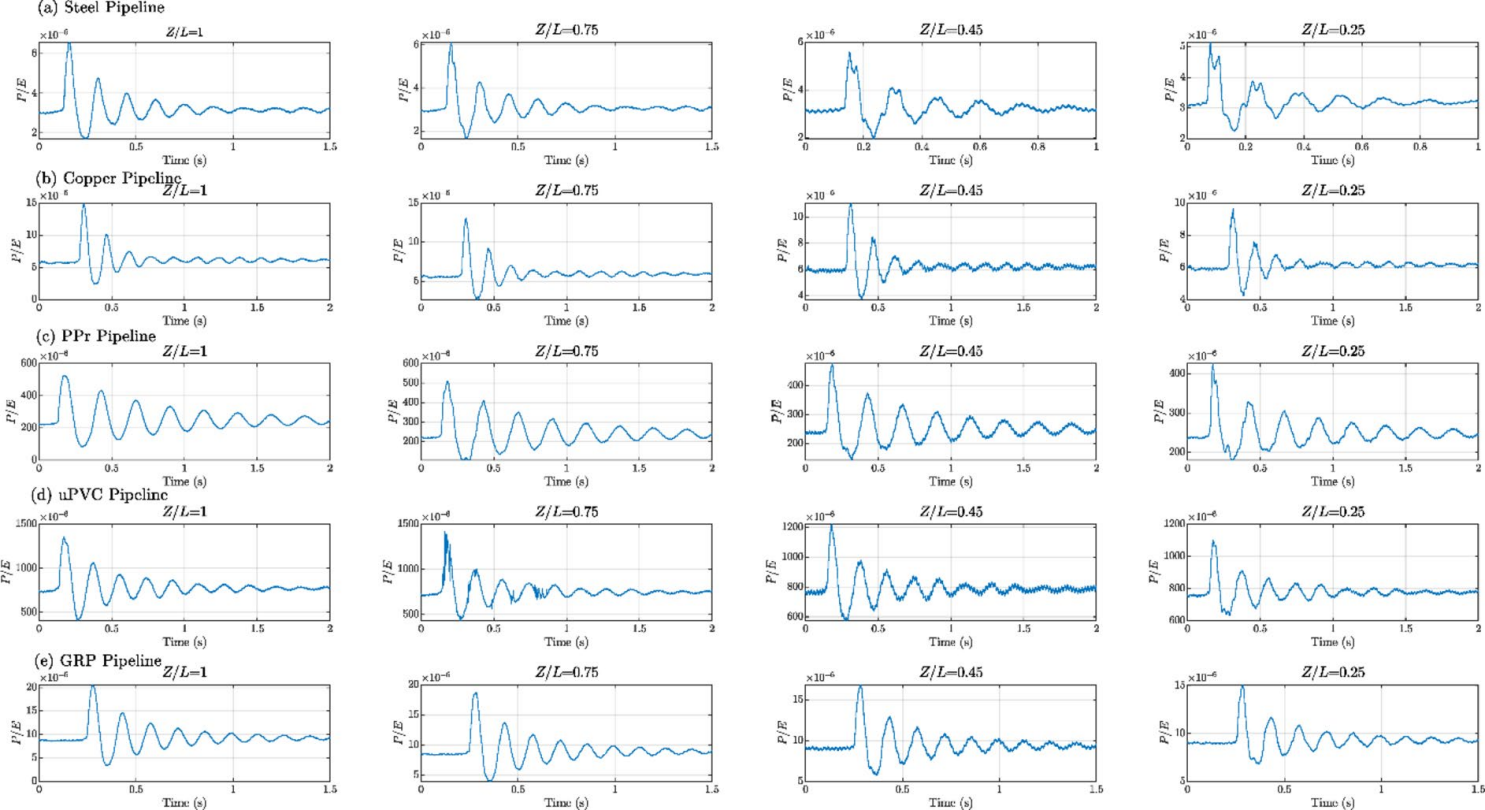
Experimental results of transient pressures ratio (*P/E*) measured at four specific locations for a pump flow rate of 50 L/min and a pressure of 6.9 bar: (**a**) Galvanized Steel, (**b**) Copper, (**c**) Polypropylene Random Copolymer (PPR), (**d**) Unplasticized Polyvinyl Chloride (uPVC), and (**e**) Glass Reinforced Plastic (GRP) pipelines.

**Fig. 13:**
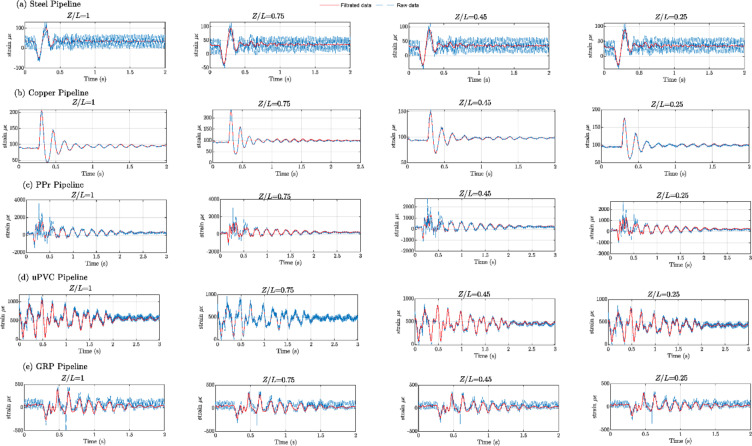
Experimental results displaying corresponding axial strain measurements (in microstrain, ${\mu}\epsilon$) at four distinct locations for a pump flow rate of 50 L/min and a pressure of 6.9 bar: (**a**) Galvanized Steel, (**b**) Copper, (**c**) Polypropylene Random Copolymer (PPR), (**d**) Unplasticized Polyvinyl Chloride (uPVC), and (**e**) Glass Reinforced Plastic (GRP) pipelines.

**Fig. 14:**
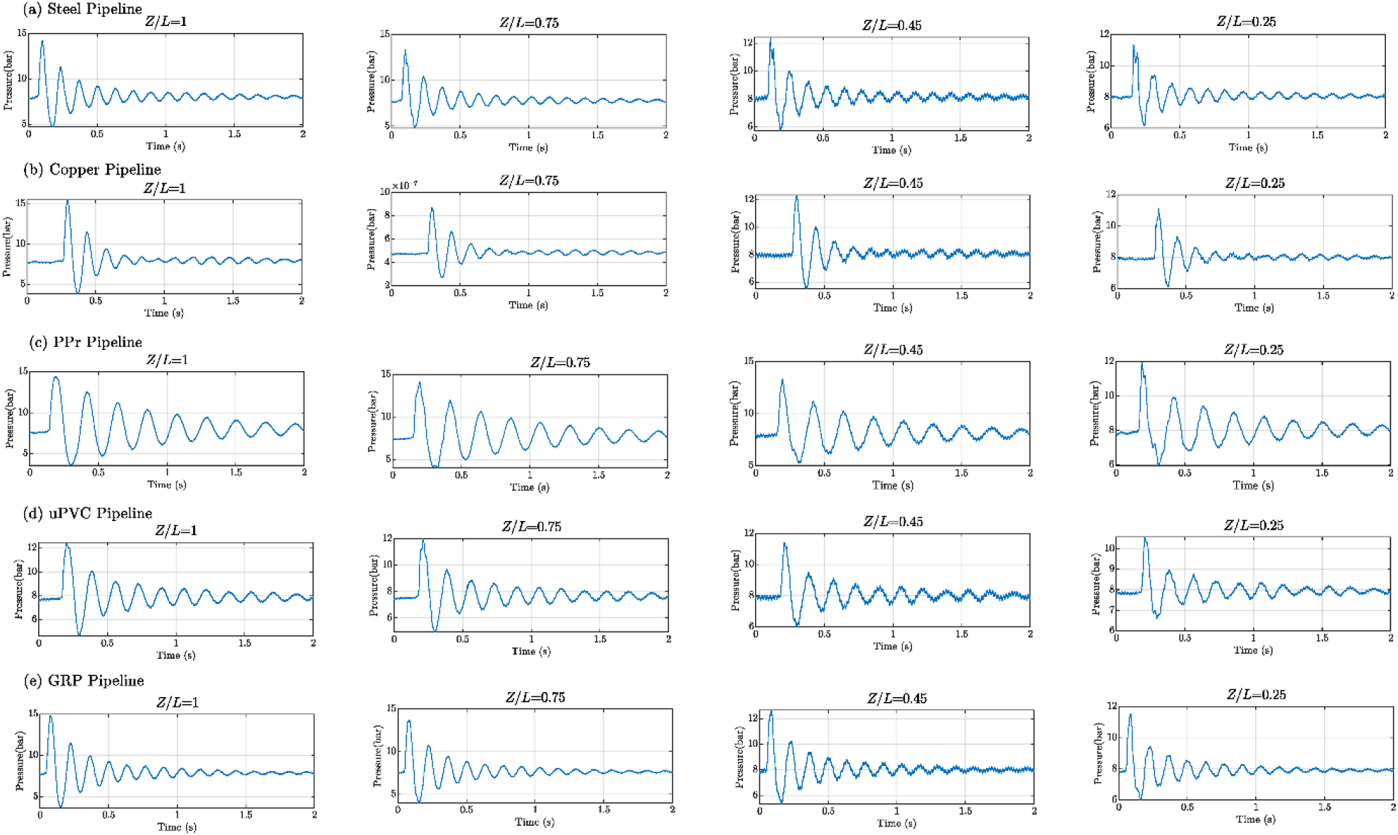
Experimental results of transient pressure ratio(*P/E*) measured at four specific locations for a pump flow rate of 40 L/min and a pressure of 8.3 bar: (**a**) Galvanized Steel, (**b**) Copper, (**c**) Polypropylene Random Copolymer (PPR), (**d**) Unplasticized Polyvinyl Chloride (uPVC), and (**e**) Glass Reinforced Plastic (GRP) pipelines.

**Fig. 15:**
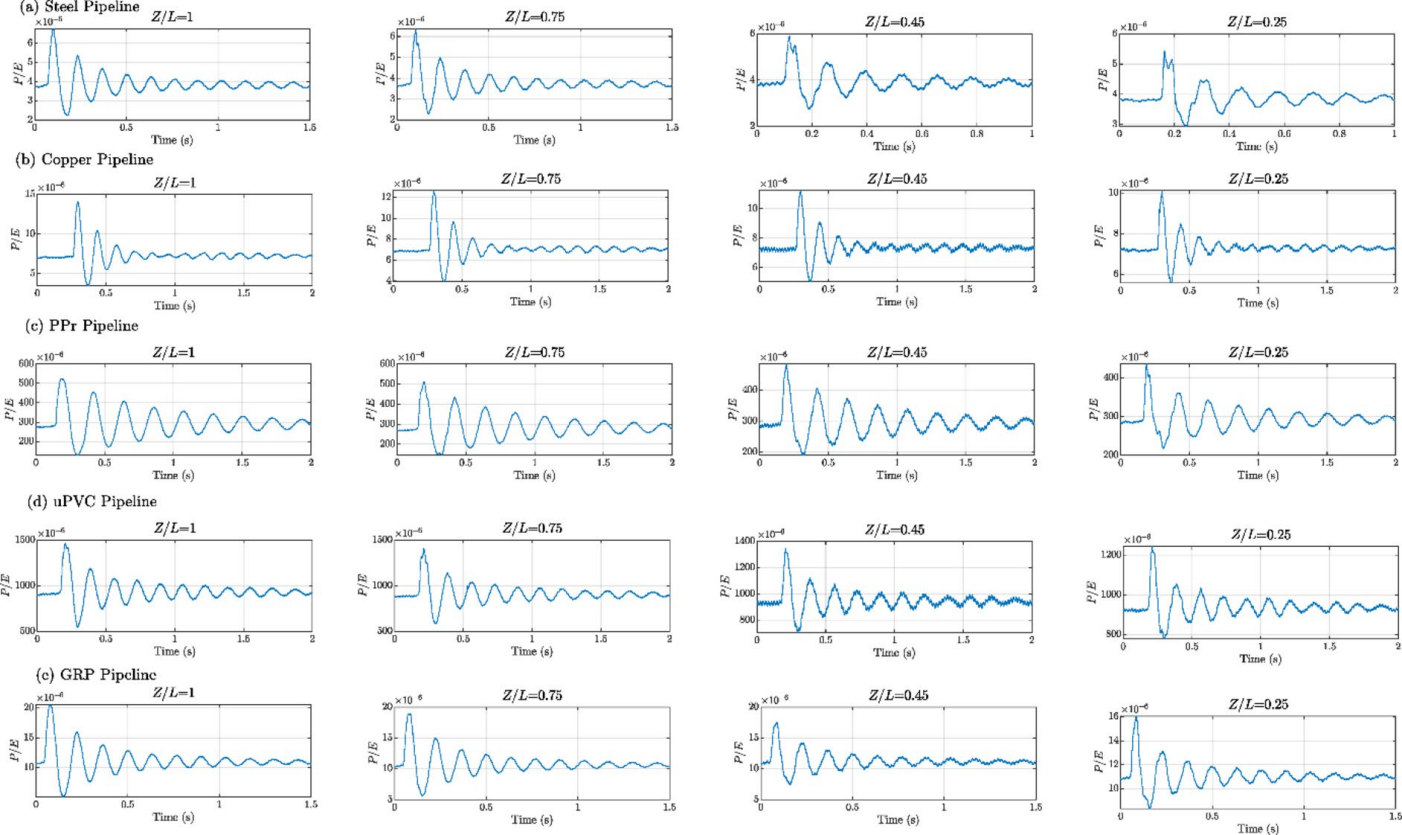
Experimental results of transient pressure ratio(*P/E*) measured at four specific locations for a pump flow rate of 40 L/min and a pressure of 8.3 bar: (**a**) Galvanized Steel, (**b**) Copper, (**c**) Polypropylene Random Copolymer (PPR), (**d**) Unplasticized Polyvinyl Chloride (uPVC), and (**e**) Glass Reinforced Plastic (GRP) pipelines.

**Fig. 16:**
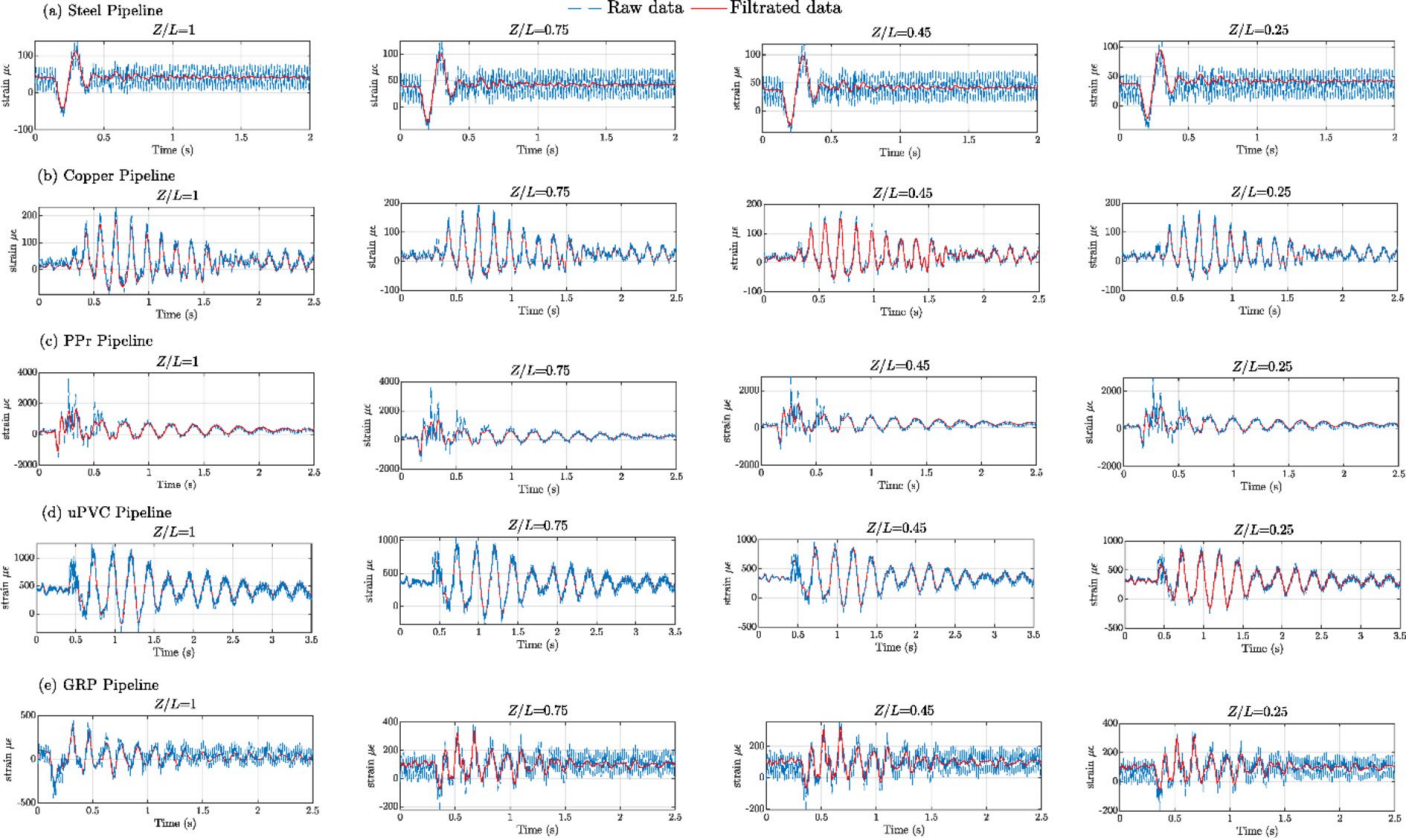
Experimental results displaying corresponding axial strain measurements (in microstrain, ${\mu}\epsilon$) at four distinct locations for a pump flow rate of 40 L/min and a pressure of 8.3 bar: (**a**) Galvanized Steel, (**b**) Copper, (**c**) Polypropylene Random Copolymer (PPR), (**d**) Unplasticized Polyvinyl Chloride (uPVC), and (**e**) Glass Reinforced Plastic (GRP) pipelines.

Figures 8, 9, 10, 11, 12, 13, 14, 15 and 16 present the outcomes of the experimental tests conducted to investigate the WH pressure and longitudinal strain in the tested pipelines. These figures visually depict the WH pressure, and the corresponding strain measured at four distinct locations for the five pipe materials (Galvanized Steel, Copper, PPr, uPVC, and GRP) employed in this study. The first column of these figures represents the WH pressure measured at a normalized distance of (*Z/L* = 1), followed by subsequent columns denoting (*Z/L*) values of (0.75), (0.45), and (0.25). Specifically, Figs. 8 and 11, and 14 exhibit the experimental pressure measurements for each pipe material. In contrast, Figs. 10 and 13, and 16 present the corresponding strain measurements for each pipe material, following the same columnar arrangement.

Lastly, Figs. 9 and 12, and 15 show the dimensionless *P/E* values associated with each pipe material. The first column of these figures displays the dimensionless WH pressure divided by the modulus of elasticity of the respective piping material at (*Z/L*).

In appendix A it is shown experimental comparison between the results of transient pressures and strain measured at four specific locations and for the five pipeline materials for a pump flow rate of 60 L/min and a pressure of 5.5 bar.

Reference to Figs. 8, 9, 10, 11, 12, 13, 14, 15 and 16, the followings are the most critical observations drawn from the different cases of experimental work.

### First case

At 60 L/min pump flow rate and 5.5 bar pressure, the water hammer test was first performed on a steel pipeline at four locations along it, the pressure at transducer P(IV), *Z/L* = 1 in got its maximum amplitude of 13.01 bar and the corresponding maximum strain value is 89.68$\:{\upmu\:}\epsilon\:$. The maximum amplitude of pressure in the coper test line was 15.48 bar and strain 187.7 $\:{\upmu\:}\epsilon\:$ at the location P. (IV) *Z/L* = 1. The transient pressure in the copper pipeline increased by 17.63% and 162% for the corresponding strain. The greatest transient pressure in the PPr pipelines was 13.20 bar and strain 1496.76 $\:{\upmu\:}\epsilon\:$. It was almost equal to the steel pipeline’s maximum transient pressure but regarding the corresponding strain it is equal to more than 16 times of corresponding strain for the steel pipeline. The greatest transient pressure in the uPVC pipelines was 10.00 bar and strain 843 $\:{\upmu\:}\epsilon\:$. It was 22.3% less than the steel pipeline’s maximum amplitude of pressure, and the corresponding strain. is equal to more than 9 times of the corresponding strain for the steel pipeline; the greatest transient pressure in the GRP pipelines was 14.50 bar and strain 383.21 $\:{\upmu\:}\epsilon\:$. It was exceeding the steel pipelines by 5.5% for maximum amplitude transient pressure, but regarding the corresponding strain it is equal to more than 4 times of the corresponding strain for steel pipeline.

### Second case

At the second test was the pump flow rate of 50 L/m and 6.9 bar pressure. the pressure at the last transducer in the steel pipeline reached its maximum value of 13.80 bar and the maximum strain value is 104.73 µε. For the copper pipeline the maximum transient pressure was 16.34 bar and strain 205.7 µε at the location closed to the solenoid valve. The transient pressure in the copper pipeline increased by 15.65% and more than three times for the corresponding strain. For the PPr pipeline the greatest transient pressure was 14.43 bar and strain 1619.12 µε. It was less than the steel pipeline’s maximum transient pressure by 5% and the strain is more than 15 times compared with the strain for steel pipeline. The greatest transient pressure in the uPVC pipelines was 12.48 bar and strain 1119.49 µε. It was 12.4% less than the steel pipeline’s maximum transient pressure as well as the corresponding strain. It is more than 10 times of strain in the steel pipeline. Finally, for the GRP pipelines the greatest transient pressure was 14.51 bar and strain 383.69 µε. And this is 5% more than the steel pipeline’s maximum transient pressure and the strain is more than 3 times the strain for the steel pipeline.

### Third case

At the final test with a pump flow rate of 40 L/m and 8.3 bar pressure. When the valve closure, the water hammer pressure at the last pressure transducer closed to the solenoid valve in the steel pipeline is the maximum value of 14.20 bar and the corresponding maximum strain value is 111.75 µε. The maximum water hammer pressure in the coper pipeline test was 15.491 bar and strain 185.65 µε at the location closed to the solenoid valve. The transient pressure in the copper pipeline increased by 8.3% and more than 166% for the corresponding strain. The greatest transient pressure in the PPr pipelines was 14.43 bar and strain 1619.12 µε. It was less than the steel pipeline’s maximum transient pressure by 5% but regarding the corresponding strain it is around 15 times of corresponding strain for steel pipeline. The greatest transient pressure in the uPVC pipelines was 12.48 bar and strain 1119.49 µε. It was 12.1% lower than the steel pipeline’s maximum transient pressure, and also the corresponding strain. It is equal to more than 10 times of corresponding strain for steel pipeline. The maximum transient pressure in the GRP pipelines was 14.80 bar and strain 393.56 µε. It was 4% more than the steel pipeline’s maximum transient pressure and the corresponding strain. It is around 3.5 times of the corresponding strain for steel pipeline.

## Numerical analysis

Unsteady state analysis of flow in pipes can utilize either an elastic or viscoelastic model, depending on the pipe material. The elastic model is simpler and suitable for predicting WH pressure fluctuations in elastic pipes, such as those made of steel and copper. In contrast, the viscoelastic model is more challenging and can be applied to examine WH effects in both elastic and viscoelastic pipes. The choice between these models is critical to accurately predict the dynamic behavior of fluid flow under varying conditions. Elastic materials typically exhibit a linear relationship between stress and strain, allowing for straightforward calculations of wave propagation speeds and pressure fluctuations using established hydraulic equations. However, for materials that display viscoelastic characteristics, such as certain plastics or composites, the behavior becomes more complex.

In viscoelastic models, both time-dependent strain and stress responses are considered, accounting for phenomena such as creep and relaxation. These models are particularly useful when examining systems subjected to rapid changes in flow, where the time-dependent properties of the pipe material significantly influence pressure surges and energy dissipation.

When simulating WH in viscoelastic pipes, it may be necessary to incorporate additional parameters, such as the material’s relaxation modulus and the damping coefficient, which describe the rate at which the material returns to its original shape after deformation. Numerical methods, like finite difference and finite element analysis, often facilitate these simulations, allowing engineers to predict potential peak pressures and assess the risk of structural failure or fatigue over time.

Ultimately, selecting an appropriate model hinges on the specific application and material characteristics. Understanding the distinctions between elastic and viscoelastic behaviors not only aids in design considerations but also enhances the reliability and longevity of piping systems in various engineering contexts. Thus, regardless of the model applied, comprehensive analysis of the materials involved and the conditions of flow remains paramount in effective pipeline management and infrastructure safety.

### Numerical analysis of elastic pipes

#### Governing equations

The numerical analysis of WH in the pipeline has been conducted using the method of characteristics (MOC) with the Modified Instantaneous Acceleration Based (MIAB) approach. An unsteady friction model (Brunone model) has been used in the analysis. The propagation of the pressure wave on the pipeline, based on the classical WH equations, has been investigated to study the transient pressure wave propagation. The analysis involves two fundamental equations, namely Eqs. (16–17), which are derived from the continuity and momentum equations of the fluid in the direction of piping. These equations form the basis for understanding the behavior of the fluid and the dynamics of the pressure wave during WH events.

16 $$\:{H}_{x}+\frac{1}{g}{V}_{t}+{h}_{f}=0$$

17 $$\:{H}_{t}+\frac{{a}^{2}}{g}{V}_{x}=0$$

where, the subscripts *x* and *t* denote the partial derivatives with respect to spatial distance and temporal progression, respectively. The term *H (*m) signifies the piezometric head, while *x* (m) and *t* (s) are used to represent the variables of distance and time, respectively. The flow velocity is symbolized by *V (*m/s), whereas the WH wave propagation speed is represented by *a* (m/s). The gravitational constant is denoted by *g* (m/s2), and *hf* symbolizes the friction head loss per unit length. The wave speed for WH is calculated as follows:

The Method of Characteristics (MOC) is employed to solve the continuity and momentum equations (Eqs. 16–17), resulting in the derivation of the following ordinary equations:

18 $$\:{C}^{+}:\left\{\begin{array}{c}\lambda\:\frac{dH}{dt}+\left(1+{k}_{t}\right)\frac{dV}{dt}+\frac{fV\left|V\right|}{2d}=0\\\:\frac{dx}{dt}=\frac{2a}{-\phi\:{k}_{x}+\sqrt{{{k}_{x}}^{2}+4(1+{k}_{t})}}\end{array}\right.\:\:$$

19 $$\:{C}^{-}:\left\{\begin{array}{c}\lambda\:\frac{dH}{dt}+\left(1+{k}_{t}\right)\frac{dV}{dt}+\frac{fV\left|V\right|}{2d}=0\\\:\frac{dx}{dt}=\frac{-2a}{\phi\:{k}_{x}+\sqrt{{{k}_{x}}^{2}+4(1+{k}_{t})}}\end{array}\right.$$

where $\:{k}_{t}$ is the Friction coefficient related to temporal derivative and $\:{k}_{x}$ is Friction coefficient related to spatial derivative. A new coefficient, *ϕ*, is introduced to modify the equation and ensure the correct sign of the damping for each valve position in the system.

**Fig. 17:**
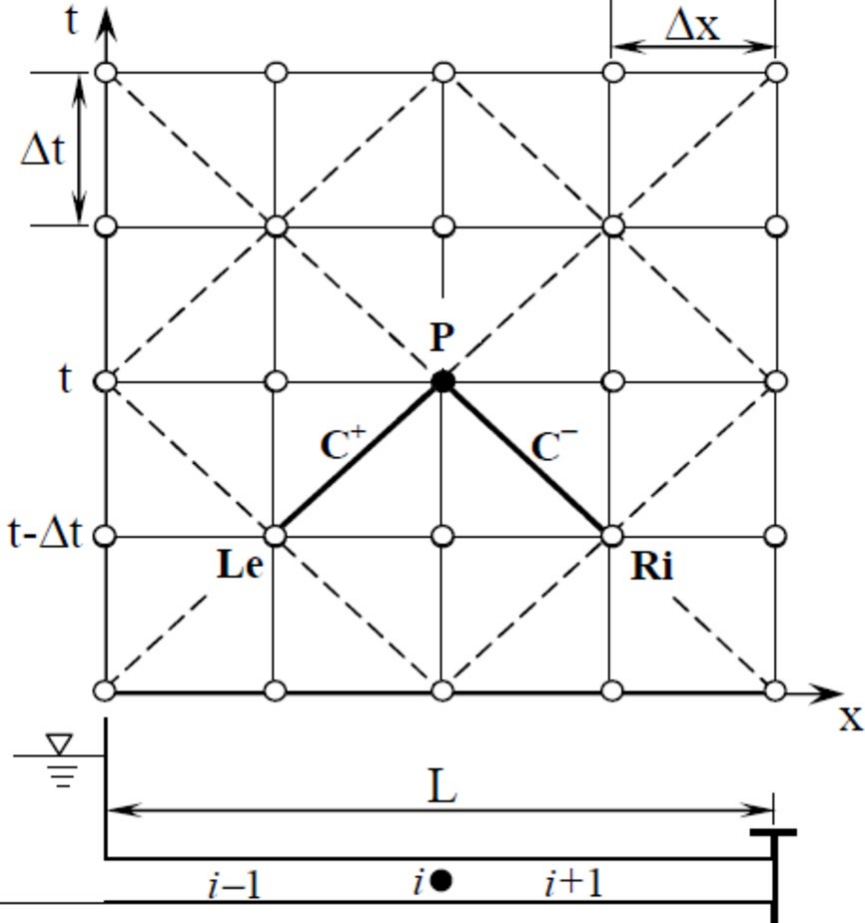
A schematic diagram illustrating the application of the method of characteristics to determine the trajectory of WH waves in the x − t plane.

Equations (18–19) were solved along characteristic lines to obtain the two unknown variables at point *P*′ by using the boundary and initial conditions, as shown in Fig. 17^68^.

20 $$\:{C}^{+}==\:\:\lambda\:=\frac{-\phi\:{k}_{x}+\sqrt{{{k}_{x}}^{2}+4(1+{k}_{t})}}{2a/g}$$

21 $$\:{C}^{-}==\:\:\lambda\:=\frac{-\phi\:{k}_{x}-\sqrt{{{k}_{x}}^{2}+4(1+{k}_{t})}}{2a/g}$$

22 $$\:{C}^{+}==\:{H}_{P}={H}_{A}-{B}^{+}\left({Q}_{P}-{Q}_{A}\right)-R{Q}_{A}\left|{Q}_{A}\right|$$

23 $$\:{C}^{+}==\:{H}_{P}={H}_{A}-{B}^{-}\left({Q}_{P}-{Q}_{A}\right)+R{Q}_{B}\left|{Q}_{B}\right|$$

24 $$\:{B}^{+}=2\frac{a}{gA}\frac{(1+{k}_{t})}{-\phi\:{k}_{x}+\sqrt{{{k}_{x}}^{2}+4(1+{k}_{t})}}$$

25 $$\:{B}^{-}=2\frac{a}{gA}\frac{(1+{k}_{t})}{\phi\:{k}_{x}+\sqrt{{{k}_{x}}^{2}+4(1+{k}_{t})}}$$

26 $$\:R=f\frac{\varDelta\:x}{2gd{A}^{2}}$$

The wave speed adjustment technique^61^ was employed to adjust the numerical wave speed with the experimental wave speed using the wave speed variation parameter $\:{{\eta}}^{{j}},\:\text{s}\text{u}\text{c}\text{h}\:\text{t}\text{h}\text{a}\text{t}:\:{{\stackrel{\sim}{\varvec{a}}}_{0}}^{{j}}=\left(1+{{\eta}}^{{j}}\right){{{a}}_{0}}^{{j}}\:$

Table 3 lists the parameters used in the numerical analysis for the different materials.

**Table 3:** WH wave speed adjustment parameters.

Pipe Material	$\:{{\eta}}^{j}$	$\:{{\varvec{a}}_{0}}^{\varvec{j}}$	$\:{{\stackrel{\sim}{\varvec{a}}}_{0}}^{\varvec{j}}$
	%	(m/s)	(m/s)
galv St	15	1431.84	1217
Cu	15	1224.20	1040.64
PPR	15	475.65	404.30
uPVC	15	647.70	550.55
GRP	15	1119.47	951.55

where $\:{{\stackrel{\sim}{\varvec{a}}}_{0}}^{\varvec{j}}$ the adjusted wave speed, and $\:{{\varvec{a}}_{0}}^{\varvec{j}}$ is the original wave speed based on Eq. 17.

The input parameters for the numerical models are the same as those used in the experimental work Table 1.

### Numerical analysis of viscoelastic pipes

The distinctive structure of polymers, which behave as a strain reaction to applied stress, underlies their unique properties. Each polymer molecule resembles a flexible thread capable of continually altering its shape by curling and twisting in response to energy input^67^. This intricate structure results in viscoelastic effects, where the macroscopic strain is accompanied by complex molecular deformations. As a result, polymers respond to applied loads in a fundamentally different way compared to elastic materials such as steel^68^.

#### Governing equations

The governing equations of motion for viscoelastic pipes under WH transients can be derived from momentum conservation principles, incorporating viscoelastic terms. The continuity and momentum equations are equation:

27 $$\:{V}_{x}+\frac{g}{{a}^{2}}{H}_{t}+2\frac{\partial\:{\epsilon\:}_{\varphi\:}}{\partial\:t}=0\:\:$$

28 $$\:{V}_{x}+\frac{g}{{a}^{2}}{H}_{t}+2\frac{\partial\:{\epsilon\:}_{\varphi\:}}{\partial\:t}=0\:\:$$

29 $$\:{\epsilon\:}_{\varphi\:}=(1-{v}^{2})({\sigma\:}_{\varphi\:}\times\:\text{d}J)$$

Where $\:{\epsilon\:}_{\varphi\:}$ is the hoop strain,$\:\:{\sigma\:}_{\varphi\:}$ is the hoop stress in Pa, $\:v$ is the Poisson’s ratio, and $\:J$ stands for creep compliance function in $\:{{P}_{a}}^{-1}$.

These modified equations provide a more accurate representation of transient behavior in viscoelastic pipes compared to traditional elastic models. The MOC is used to solve these equations due to the complexities introduced by viscoelastic behavior which produces WH compatibility equations valid along characteristic lines in the distance (X)–time (*t*) plane.

Methodology for selecting creep parameters and the code used in this section was obtained through personal communication with the author of reference^28^.The input parameters for the unplasticized polyvinyl chloride (uPVC) pipe are as follows: pipe length of 12 m, inner diameter of 27.2 mm, outer diameter of 32 mm, modulus of elasticity of 3.38 GPa, and a friction coefficient of 0.02.

### Comparison of numerical and experimental results

**Fig. 18:**
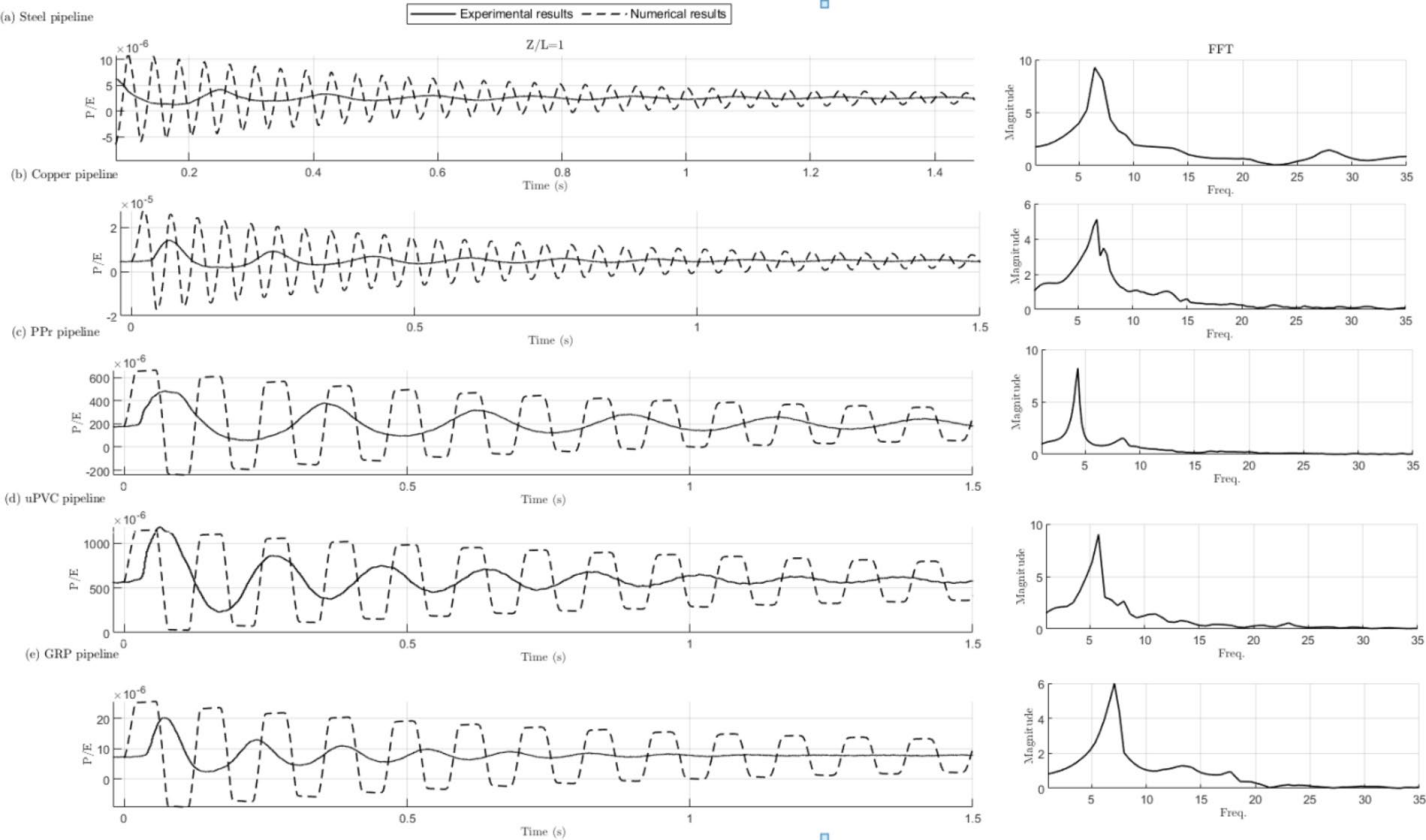
Comparison between experimental and numerical dimensionless pressure results with time of water hammer (WH) at a steady-state flow rate of 50 L/min and a steady-state pressure of 6.9 bar at the location *Z/L* = 1 is presented. The corresponding FFT subfigures for experimental pressure results are presented on the right side for different pipeline materials: (**a**) galv St (**b**) Cu (**c**) PPr (**d**) uPVC (**e**) GRP.

Figure 18 presents both theoretical and experimental results of the water hammer phenomenon. The theoretical results for the metallic and composite pipes were obtained using an elastic model, while the theoretical results for PPR and PVC pipes were derived using the viscoelastic model referenced in^28^.

Figure 18 results exhibit a notable deviation between theoretical and experimental outcomes. The figure results indicate that the frequency of the measured pressure signal is considerably lower than the corresponding analytical values.

The authors assert that the primary cause behind this deviation is the greater flexibility of the present pipeline structure as it was observed that the pressure waves, particularly in steel and PPR, matched one of the pipe’s natural frequency waves. In addition, the use of supports to just level the real system under investigation. Conversely, the theoretical framework employed in this study assumes rigid pipe-ground connections, which are not compatible with the current setup. Thus, the readings during the experiment may reflect greater effect of the system natural frequency waves rather than water hammer effects.

Furthermore, the frequency analysis of the experimental scenario reveals the presence of two frequencies in certain cases, such as the steel and PPr. Significantly, for steel the first frequency, displaying a higher amplitude, aligns more closely with the system’s natural frequency, while the second frequency aligns more closely with the theoretical WH frequency. This observation offers a compelling explanation for the observed deviation between the experimental and analytical frequency responses, suggesting that the system’s behavior is governed by a combination of its inherent resonance and the transient pressure fluctuations associated with WH phenomena.

**Fig. 19:**
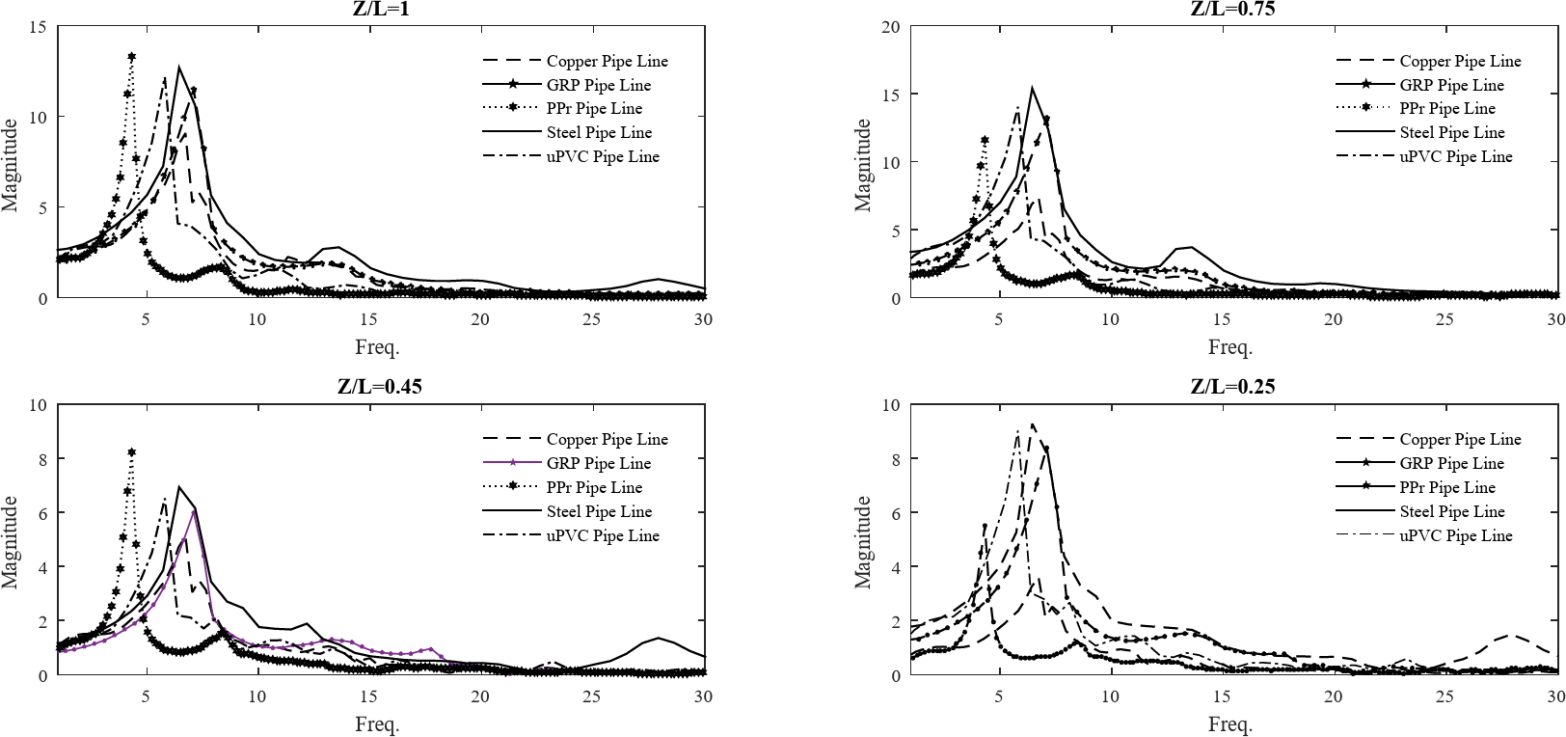
FFT analysis for the five pipe materials at four locations on the pipeline.

Figure 19 exhibits the compression of FTT analysis for the five pipe materials. The water hammer event is often exciting the natural frequency of the system, especially since the system is flexible rather than rigid. Knowing the precise natural frequency value is critical, as it allows us to properly interpret the Fast Fourier Transform (FFT) analysis. The flexible nature of the system means the water hammer can resonate with the natural frequency, potentially leading to significant damage if not properly addressed. The frequency analysis of the experimental scenario unveils the presence of dual frequencies in specific cases, notably observed in the steel and PPr pipe material combinations. Notably, the primary frequency, characterized by a higher amplitude, exhibits a closer alignment with the system’s natural frequency, while the secondary frequency demonstrates a closer correspondence with the theoretical WH frequency. This observation provides a compelling rationale for the observed disparities between the experimental and analytical frequency responses, indicating that the system’s dynamics are influenced by a synergistic interplay between its intrinsic resonance characteristics and the transient pressure fluctuations inherent in WH phenomena.

**Fig. 20:**
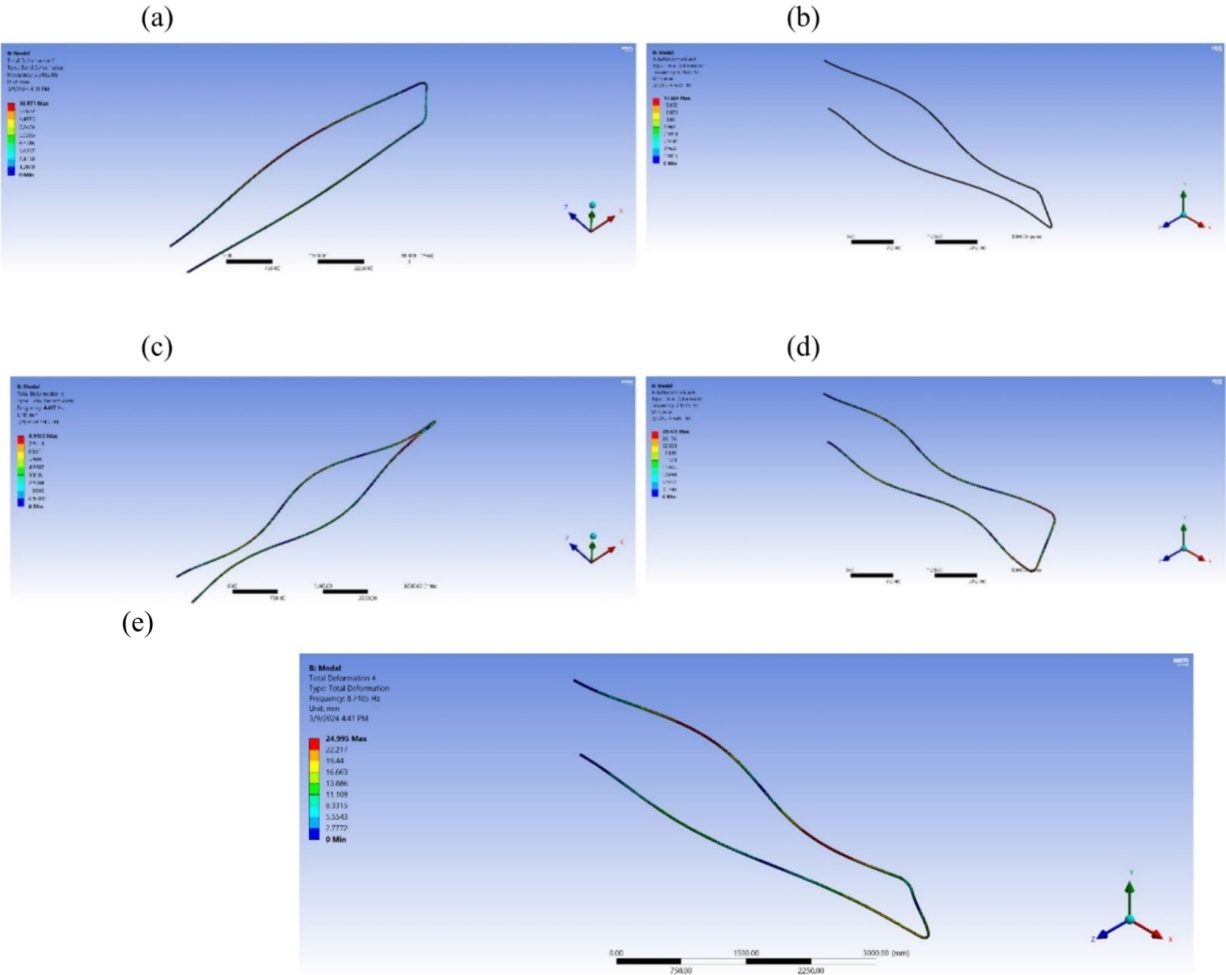
Natural frequency (**a**) Steel pipeline. (**b**) Copper pipeline. (**c**) PPr pipeline. (**d**) uPVC pipeline. (**e**) GRP Pipeline for Z/L = 1.

ANSYS 19.2 software was utilized. A detailed model was created to extract natural frequencies and mode shapes for each pipe material., Fig. 20 (a) depicts the natural frequency of the steel pipeline as 5.34 Hz, matching the numerical analysis value presented in Fig. 18 (Z/L = 1). Figure 20 (b) depicts the natural frequency of the copper pipeline as 6.36 Hz, matching the numerical analysis value presented in Fig. 18 (Z/L = 1). Figure 20 (c) depicts the natural frequency of the PPr as 4.48 Hz, matching the numerical analysis value presented in Fig. 18 (Z/L= 1). Figure 20 (d) depicts the natural frequency of the uPVC pipeline as 6.36 Hz, closed to the numerical analysis value presented in Fig. 18 (Z/L = 1). Figure 20 (e) depicts the natural frequency of the GRP pipe line as 8.7 Hz which is close to the numerical analysis value presented in Fig. 18 (Z/L = 1).

Modal analysis was conducted using ANSYS software to investigate the natural frequencies and mode shapes of the pipeline system under water hammer conditions. The ANSYS model incorporated the geometric details, material properties, and boundary conditions of the pipeline, utilizing SOLID185 for solid elements and SHELL181 for shell elements to accurately capture the structural response. The obtained natural frequencies from ANSYS modal analysis were compared to those derived from the Method of Characteristics (MOC) model to validate the accuracy of the simulation approach. The results revealed the natural frequencies for different pipe materials, such as steel, copper, PPr, and uPVC, highlighting the system’s vibrational characteristics. Understanding the natural frequencies is crucial in water hammer analysis, as the pressure waves can excite the system’s resonant frequencies, particularly in flexible systems. This knowledge aids in interpreting the Fast Fourier Transform (FFT) analysis of pressure signals, providing insights into potential resonance issues and system behavior during water hammer events. The modal analysis using ANSYS serves as a valuable tool in predicting and mitigating the effects of water hammer in pipeline systems, characterized by eight nodes and six degrees of freedom at each node. The element size was set to 2 mm, resulting in a total of 3,024,000 elements for a pipe length of 12 m.

**Fig. 21:**
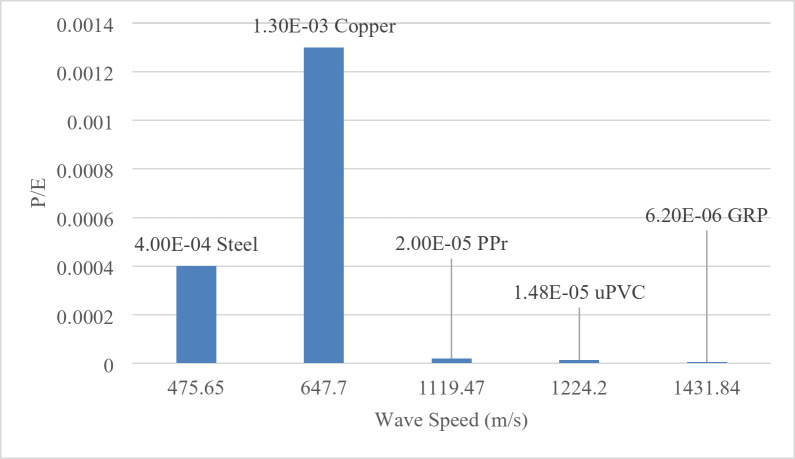
P/E versus the wave speed.

Figure 21 provided insightful correlation between the maximum pressure-to-modulus ratio (P/E) and the corresponding wave speeds for various pipe materials. This correlation offers a predictive relationship used to estimate the maximum pressure experienced during a WH event based on the material properties of the pipe.

Wave speed and *P/E* relationship: The plot shows a clear inverse correlation between the wave speed and the maximum P/E ratio. As the wave speed increases, the maximum P/E ratio decreases. This trend is observed across the different pipe materials, indicating a fundamental relationship between these two parameters.

Highest P/E ratio: The uPVC pipe material exhibits the highest maximum P/E ratio of 0.0013, which is significantly higher than the other materials. This suggests that uPVC pipes are more susceptible to experiencing high pressure spikes relative to their modulus of elasticity during water hammer events.

Lowest *P/E* ratio: In contrast, the steel pipe material has the lowest maximum *P/E* ratio of 0.0000062, indicating that it can withstand much higher pressures without exceeding the material’s elastic limit.

Predictive relationship: The clear correlation between wave speed and maximum P/E ratio provides a valuable predictive tool for engineers and designers. By knowing the material properties of a pipe, they can estimate the potential maximum pressure that the pipe will experience during a WH event, which is crucial for designing robust and reliable piping systems.

This plot and the associated discussion represent a significant contribution to the field of WH analysis in viscoelastic pipes. The ability to predict the maximum pressure based on material properties adds a novel aspect to the research and enhances the practical applicability of the findings. The insights gained from this analysis can inform the selection of appropriate pipe materials and the design of mitigation strategies to prevent or minimize the detrimental effects of WH in various engineering applications.

## Discussions

This study aims to comprehensively investigate the impact of different pipe materials on WH phenomena using a combination of experimental measurements and numerical simulations. The experimental measurements are performed on a dedicated test rig that simulates a pressure pipeline, while the numerical simulations utilize the method of characteristics (MOC). The findings of this study underscore the criticality of selecting the appropriate pipe material, as it exerts a substantial influence on the occurrence and severity of WH events. Furthermore, the study highlights the significance of considering fluid-structure interaction when analyzing WH in pressure pipelines. The interplay between the fluid and the pipe structure plays a pivotal role in the behavior of the system and should be meticulously taken into account during the pipeline design phase.

This study systematically investigates the failure analysis of different pipe materials under WH conditions. The von Mises criterion is employed as the failure theory for all pipe materials except for Glass Reinforced Plastic (GRP), where the Tsai-Wu criterion is utilized. Through the utilization of these failure criteria, this research provides significant contributions towards understanding the structural integrity of diverse pipe materials when subjected to WH scenarios. Furthermore, it assesses the suitability of these materials for application in pressure pipelines. The von Mises stress is determined by the expression

30 $$\:{\sigma\:}_{vM}={\left[\frac{({\sigma\:}_{1}-{{\sigma\:}_{2})}^{2}+({\sigma\:}_{2}-{{\sigma\:}_{3})}^{2}+({\sigma\:}_{3}-{{\sigma\:}_{1})}^{2}}{2}\right]}^{\frac{1}{2}}$$

where the principal stresses in the three-dimensional space are defined as follows:

31 $$\:{\sigma\:}_{1}=\frac{{\sigma\:}_{l}+{\sigma\:}_{\theta\:}}{2}+\sqrt{{\left(\frac{{\sigma\:}_{l}-{\sigma\:}_{\theta\:}}{2}\right)}^{2}+{{\tau\:}_{xy}}^{2}}$$

32 $$\:{\sigma\:}_{2}=\frac{{\sigma\:}_{l}+{\sigma\:}_{\theta\:}}{2}-\sqrt{{\left(\frac{{\sigma\:}_{l}-{\sigma\:}_{\theta\:}}{2}\right)}^{2}+{{\tau\:}_{xy}}^{2}}$$

33 $$\:{\sigma\:}_{3}={\sigma\:}_{r}$$

In the absence of shear stress (*τxy* = 0), the principal stresses simplify to:

34 $$\:{\sigma\:}_{1}=\:{\sigma\:}_{l}\:,\:\:{\sigma\:}_{2}=\:{\sigma\:}_{\theta\:}\:,\:\:{\sigma\:}_{3}=\:{\sigma\:}_{r\:}$$

resulting in the von Mises stress:

35 $$\:{\sigma\:}_{vM}={\left[\frac{({\sigma\:}_{l}-{{\sigma\:}_{\theta\:})}^{2}+({\sigma\:}_{\theta\:}-{{\sigma\:}_{r})}^{2}+({\sigma\:}_{r}-{{\sigma\:}_{l})}^{2}}{2}\right]}^{\frac{1}{2}}$$

The maximum stress occurs at the inner cylinder surface for r = Ri. The Factor of Safety (FOS) is computed as:

36 $$\text{Factor of Safety}, (FOS)=\:\frac{{\varvec{\sigma\:}}_{\varvec{y}}}{{\sigma\:}_{vM}}$$

The calculation of the factor of safety using the Tsai-Wu failure criterion for GRP pipelines is outlined as follows.

The tensile and compressive strengths of composite materials in the longitudinal and transverse directions, denoted as *F*1*t*, *F*1*c*, *F*2*t*, and *F*2*c*, can be determined using the following equations^62^:

37 $$\:{\varvec{F}}_{1\varvec{t}\:}=\:{F}_{ft}\left({V}_{f}+{V}_{m}\frac{{E}_{m}}{{E}_{f}}\right)$$

38 $$\:{\varvec{F}}_{1\varvec{c}\:}=\:0.5\:{F}_{1t\:}$$

39 $$\:{\varvec{F}}_{2\varvec{t}\:}=\:{{V}_{m}F}_{mt}$$

40 $$\:{\varvec{F}}_{2\varvec{c}\:}=\:{{V}_{m}F}_{mc}$$

where *Ff t* represents the tensile strength of the E-glass fiber, *Fmt* and *Fmc* denote the tensile and compressive strengths of the polyester matrix, respectively. The mechanical properties of the fiber and the polyester matrix, based on the manufacturer’s data sheet, are summarized in Table 4.

**Table 4:** Mechanical properties of fiber and polyester matrix.

Mechanical properties	E-glass fiber	Polyester matrix
Young modulus (GPa), $\:{{E}}_{{f}}$& $\:{{E}}_{{m}}$	70	3.5
Shear modulus (GPa), $\:{{G}}_{{f}}$& $\:{{G}}_{{m}}$	26.89	1.32
Poisson’s ratio, $\:{{v}}_{{f}}$& $\:{{v}}_{{m}}$	0.22	0.33
Tensile strength (MPa), $\:{{F}}_{ft}\&{{F}}_{{mt}}\:$	3400	78
Compressive strength (MPa), $\:{{F}}_{{mc}}$	-	400
Density (g/cm^3^), $\:{{\rho\:}}_{f}$& $\:{{\rho\:}}_{{m}}$	2.56	1.2

The Tsai-Wu interaction parameter is given by the following equation^63^:

$$\:{A=-\frac{{{\sigma\:}_{1}}^{2}}{{{F}_{1t}F}_{1c}}\:-\frac{{{\sigma\:}_{2}}^{2}}{{{F}_{2t}F}_{2c}}\:-\frac{{{\sigma\:}_{3}}^{2}}{{{F}_{3t}F}_{3c}}+\frac{{{\tau\:}_{12}}^{2}}{{{F}_{12}}^{2}}\:\:\:+\frac{{{\tau\:}_{23}}^{2}}{{{F}_{23}}^{2}}}_{}+\frac{{{\tau\:}_{13}}^{2}}{{{F}_{13}}^{2}}+{c}_{12}\frac{{\sigma\:}_{1}{\sigma\:}_{2}}{\sqrt{{{F}_{1t}F}_{1c}{{F}_{2t}F}_{2c}}}$$

41 $$\:+{c}_{23}\frac{{\sigma\:}_{2}{\sigma\:}_{3}}{\sqrt{{{F}_{2t}F}_{2c}{{F}_{3t}F}_{3c}}}+{c}_{13}\frac{{\sigma\:}_{1}{\sigma\:}_{3}}{\sqrt{{{F}_{1t}F}_{1c}{{F}_{3t}F}_{3c}}}$$

42 $$\:B=\left(\frac{1}{{F}_{1t}}+\frac{1}{{F}_{1c}}\right){\sigma\:}_{1}+\left(\frac{1}{{F}_{2t}}+\frac{1}{{F}_{2c}}\right){\sigma\:}_{2}+\left(\frac{1}{{F}_{3t}}+\frac{1}{{F}_{3c}}\right){\sigma\:}_{3}$$

43 $$\:FOS=\left(-\frac{B}{2 A}+\sqrt{{\left(\frac{B}{2 A}\right)}^{2}+\frac{1}{A}}\right)$$

In these equations, *τij* represents the shear stress in the *ij* plane, *cij* are the Tsai-Wu coupling coefficients, *Fit* and *Fic* denote the tensile and compressive strengths of the GRP composite material in the *i* direction, where 1 corresponds to the fiber direction and 2 corresponds to the transverse direction, and *Fij* represents the shear strength in the *ij* plane. The GRP composite pipe material properties are listed in Table 5.

**Table 5:** GRP composite pipe material properties.

GRP material properties	
$\:{{E}}_{11}$ (GPa)	16.30
$\:{{E}}_{22}$ (GPa)	4.28
$\:{{v}}_{12}$	0.30
$\:{{v}}_{21}$	0.08
$\:{{{F}}_{1{t}\:}}_{}$(MPa)	791.93
$\:{{F}}_{1{c}}$(MPa)	395.96
$\:{{F}}_{2{t}\:\:}={{F}}_{3{t},\:\:\:}\:$(MPa)	63.18
$\:{{F}}_{2{c}}=\:{{F}}_{3{c},\:}\:$(MPa)	324
$\:{{G}}_{12\:}$(GPa)	1.61
Fiber volume ratio$\:\:\left(\:{{V}}_{{f}\:}\right)$	0.19
Fiber angle $\:\left({\theta\:}\right)$	57^o^

A finite element model was established using ANSYS Workbench (R19.2) with the ACP module for a GRP pipe subjected to the maximum WH pressure for each case. The SOLID185 element was used, characterized by eight nodes and six degrees of freedom at each node. The element size was set to 2 mm, resulting in a total of 25,200 elements for a pipe length of 100 mm.

Table 6 provides a summary of the experimental results related to WH and the calculated factors of safety for various pipe materials. The peak pressure of the WH, denoted as *P*max, was measured experimentally. The peak of the WH measured strain signal is represented as *ε*max. The value of *P*max was utilized to calculate the hoop stress (* σ*_*θ*_), longitudinal stresses (*σ*_*l*_), and radial stress (*σ*_*r*_), which were subsequently employed to evaluate the principal stresses. The calculated longitudinal strain at the peak pressure of WH is *ε*l. The maximum principal stress is denoted as *σ*max and was used in the calculation of the factor of safety (FOS) for each pipe material under each of the three flow and pressure conditions.

**Table 6:** Summary of experimental and theoretical results for different pipe materials and factor of safety calculation.

Pipe material	Flow rate	Flow pressure		Experimental results	Theoretical results		Factor of safety
	**(L/min)**	**(bar)**	$\:{\varvec{P}}_{\varvec{m}\varvec{a}\varvec{x}}$**(bar)**	$\:{\varvec{\epsilon\:}}_{\varvec{m}\varvec{a}\varvec{x}}$**(µε)**	$\:{\varvec{\sigma\:}}_{\varvec{m}\varvec{a}\varvec{x}}$**(MPa)**	$\:{\varvec{\epsilon\:}}_{\varvec{l}}$**(µε)**	**(FOS)**
galv St	60	5.5	13.01	89.68	4.66	48.26	60.22
	50	6.9	13.80	104.73	4.876	51.19	56.77
	40	8.3	14.20	111.75	5.02	52.68	55.17
Cu	60	5.5	15.48	187.70	14.78	221.70	5.43
	50	6.9	16.34	205.7	15.56	245.33	5.15
	40	8.3	15.49	185.65	14.73	224.35	5.43
PPR	60	5.5	13.20	1496.76	1.99	1856	7.18
	50	6.9	14.47	1625	2.35	1918	6.55
	40	8.3	14.43	1619.12	2.43	1933	6.57
uPVC	60	5.5	10.00	843	5.89	1050	6.98
	50	6.9	11.50	1047.5	6.44	1261	6.07
	40	8.3	12.48	1119.49	6.42	1321	5.59
GRP E	60	5.5	14.50	383.21	14.41	212.80	5.69
	50	6.9	14.51	388.69	14.43	212.81	5.69
	40	8.3	14.80	393.56	14.93	214.56	5.71

**Fig. 22:**
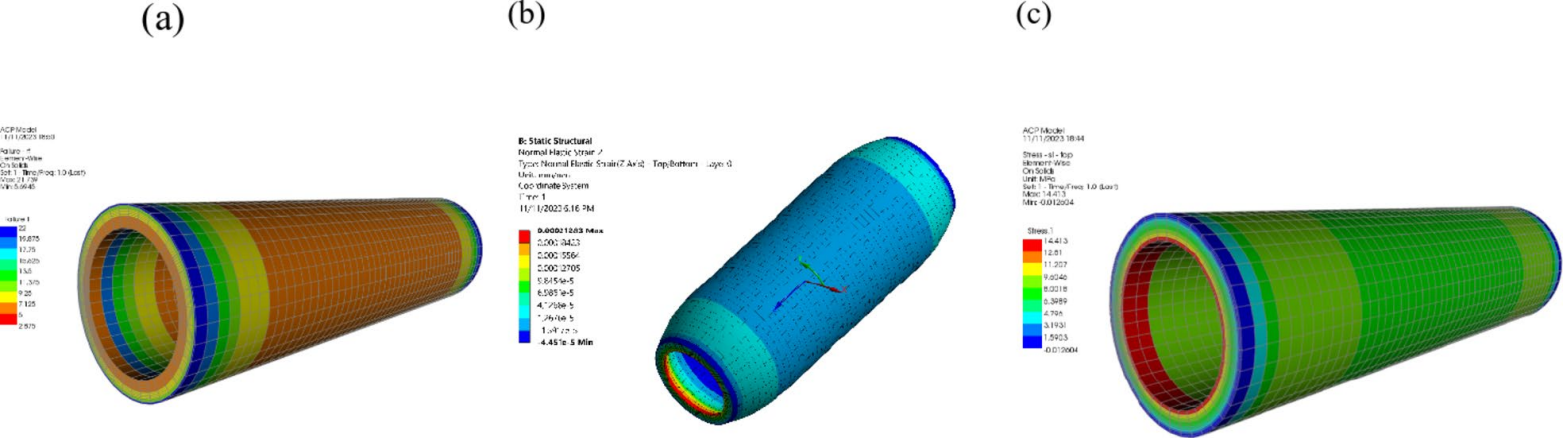
(**a**) Distribution of the factor of safety. (**b**) the longitudinal strain in the inner and outer surface. (**c**) the stress in the inner and outer surface of the GRP pipe at 14.50 bar.

 Figure 22 (a) show the distribution of the factor of safety along of test pipe, Fig. 22 (b) show the longitudinal strain in the inner and outer surface test pipe, Fig. 22 (c) show the stress in the inner and outer surface of the GRP pipe at 14.50 bar.

The safety factor for galvanized steel pipe is calculated as 60.22, copper pipe as 5.43, polypropylene (PPr) as 7.18, unplasticized polyvinyl chloride (uPVC) as 6.98, and glass-reinforced plastic (GRP) as 5.69. Based solely on the calculated safety factor values, it can be inferred that galvanized steel (galv St.) is the most suitable material for withstanding WH effects. With a safety factor of 60.22, galvanized steel can withstand significantly higher stress levels before experiencing failure or deformation. However, it is crucial to consider other factors, such as piping fixation, to make a definitive assessment.

In contrast, copper exhibits a considerably lower safety factor of 5.43 compared to galvanized steel. This suggests that copper may be more prone to failure or deformation under WH conditions, primarily due to its lower pipe thickness compared to galvanized steel. Table 6 presents the safety factors for GRP, PPr, and uPVC, which are determined as 5.69, 7.18, and 6.98, respectively. These values suggest the potential suitability of these materials for resisting WH effects. However, to ascertain the optimal material for withstanding WH, a comprehensive analysis incorporating material properties, dynamic loading behavior, and cost considerations are imperative.

Before the occurrence of a WH event, it is observed during the measurement process that the pressure values at locations *Z/L* = 1 and *Z/L* = 0.75 are lower compared to the pressure values at *Z/L* = 0.45 and *Z/L* = 0.25. This discrepancy can be attributed to the static pressure difference, approximately 1 m head, between the transducers situated in the lower region of the test pipeline near the pump and the transducers positioned in the upper region of the test pipeline. Additionally, friction losses between the liquid and the pipe wall introduce variations in pressure values at different points along the pipeline. During a WH event, the pressure near the solenoid valve at the pressure transducer *PIV*, as depicted in Fig. 8, reaches its maximum value. This high pressure occurs in the upper portion of the test line, *Z/L* = 1, due to the substantial pressure change associated with the WH. This change is significantly greater than the pressure difference resulting from the change in the levels between the upper and lower parts of the test line and the friction loss.

Discussion of potential sources of discrepancies, in comparing the numerical and experimental results of WH events, several potential sources of discrepancies were identified. Understanding these factors is crucial for interpreting the results and improving the accuracy of future simulations. The following points outline the primary sources of discrepancies observed in our study:

Material Property Variability: The viscoelastic properties of materials can vary significantly due to manufacturing processes, environmental conditions, and aging effects. In our experiments, the creep parameters were derived from specific samples, which may not fully represent the variability in material properties across different batches or installations. This variability can lead to differences in the predicted and observed responses during WH events.

Boundary conditions and system configuration: The numerical model was developed based on idealized boundary conditions and system configurations. In contrast, the experimental setup may have introduced complexities such as friction losses, fittings, and bends that were not fully accounted for in the model. These factors can alter the pressure wave propagation and attenuation characteristics, leading to discrepancies between the numerical predictions and experimental measurements.

Measurement uncertainties: Experimental measurements are subject to uncertainties due to limitations in sensor accuracy, calibration, and data acquisition methods. For instance, pressure transducers may have inherent lag or drift, affecting the recorded pressure transients. Additionally, the placement of sensors along the pipeline can influence the observed pressure and strain responses, potentially leading to misinterpretations of the data.

Transient friction effects: The numerical model may have employed a steady-state friction model, which does not account for the unsteady friction that can occur during rapid pressure changes. Experimental studies have shown that transient friction can significantly impact the damping of pressure waves and neglecting this phenomenon in the numerical simulations may contribute to discrepancies in the predicted pressure profiles.

Assumptions in the numerical Model: The numerical model relies on certain assumptions, such as linearity and homogeneity of the material properties, which may not hold true in real-world applications. For example, the assumption of constant wave speed may not accurately reflect the behavior of viscoelastic materials under varying loading conditions. These simplifications can lead to differences in the predicted behavior compared to.

In conclusion, while the numerical model provides valuable insights into the behavior of pipeline systems under transient conditions, it is essential to recognize the potential sources of discrepancies when interpreting the results. Future work should focus on refining the model by incorporating more realistic boundary conditions, accounting for material variability, and improving measurement techniques to enhance the alignment between numerical predictions and experimental observations.

## Conclusion

The objective of this study was to investigate the influence of different pipe materials on WH in pressure pipelines, employing a combination of experimental and numerical methods. The results demonstrate that the choice of pipe material significantly impacts the magnitude and duration of WH. The study encompassed the testing of five distinct materials: galvanized steel (galv St.), copper (Cu), unplasticized polyvinyl chloride (uPVC), polypropylene (PPr), and glass-reinforced plastic (GRP). The numerical simulations align with the experimental findings.

To determine the most suitable pipe material for withstanding WH, several factors must be considered. These factors encompass pipeline dimensions (such as diameter and wall thickness), material properties related to elasticity and strength (including tensile and yield strength), and the material’s ability to absorb or dampen pressure surges. Additionally, the specific application and operating conditions must be carefully evaluated.

The following conclusions can be drawn from the experimental data and theoretical results: The maximum transient pressure demonstrates close concurrence between the theoretical and experimental findings for all pipe materials and at various locations along the pipeline. However, a significant deviation arises when comparing the experimental and theoretical outcomes in terms of WH frequency. This discrepancy can be rationalized by the absence of rigid support in the current pipeline test rig.

The safety factor values indicate that galvanized steel (galv St.) exhibits the highest safety margin and may be the most suitable material for WH resistance. Nonetheless, a comprehensive analysis considering multiple factors is indispensable for making informed decisions regarding the selection of the optimal pipe material for a given application. During the experimental work, uPVC material pipelines exhibited the smallest transient peak pressure. Additionally, the highest strain was observed in PPr material pipelines. The findings indicate that WH in pressure pipelines can be influenced by the type of pipe material employed.

According to the research, the selection of suitable pipe materials for pressure pipelines is crucial in mitigating the impact of WH. Furthermore, the study provides valuable insights into the behavior of different pipe materials under dynamic loading conditions, facilitating future design considerations for pressure pipelines.

## Electronic supplementary material

Below is the link to the electronic supplementary material.

Supplementary Material 1

## Data Availability

The datasets generated during and/or analyzed during the current study are available from the corresponding author on reasonable request.

## List of symbols

${A}_{f}$: Fluid cross-sectional area (m^2^)
${A}_{p}$: Pipe cross-sectional area (m^2^)
$a$: Wave speed in the fluid (m/s)
$c$: Wave speed in the pipe wall (m/s)
$d$: Pipe inner diameter (m)
$E$: Young’s modulus of pipe materials (Pa)
$$F$$: Force (N)
$f$: Darcy Weisbach friction coefficient (–)
$$g$$: Gravitational acceleration (m/s^2^)
$G$: Shear modulus of pipe materials (Pa)
$$H$$: Hydraulic head (m)
$h$: Pipe-wall thickness (m)
${h}_{f}$: Friction losses head (m)
$I$: Area moment of inertia (m^4^)
$J$: Polar moment of inertia (m^4^)
$K$: Bulk modulus of compressibility (Pa)
$$L$$: Pipe length (m)
$$M$$: Moment (N.m)
${m}_{v}$: Valve mass (kg)
$P$: Fluid pressure (Pa)
${P}_{tank}$: Water head in the tank (m)
$$Q$$: Discharge (m^3^/s)
${R}_{i}$: Pipe inner radius (m)
${R}_{o}$: Pipe outer radius (m)
$$t$$: Time (s)
$U$: Axial pipe velocity (m/s)
$$V$$: Flow velocity (m/s)
$${V}_{r}$$: Relative velocity (m/s)
$W$: Pipe-wall radial movement (m/s)
z: Pipe axis direction (–)
$$\varepsilon$$: Strain (–)
$$v$$: Poisson’s ratio (–)
${\rho }_{f}$: Fluid Density (kg/m^3^)
${\rho }_{p}$: Pipe material Density (kg/m^3^)
$\sigma$: Axial pipe stress (Pa)
$\tau$: Shear stress fluid-pipe (Pa)
$\tau \left(t\right)$: Dimensionless number of the valve discharge coefficient (–)
$\Omega$: Boundary conditions coefficient (–)
$\gamma$: Angle between pipe axis and horizontal plane (Degree)
$\mu$: Dry friction coefficient (–)
$$\Delta t$$: Time step (S)
