# Supplementary material for: Unveiling the impact of pipe materials on water hammer in pressure pipelines: an experimental and numerical study
Source: Sci Rep. 2024 Dec 23;14:30599. doi: 10.1038/s41598-024-80853-w (PMC11666582; doi:10.1038/s41598-024-80853-w)
Supplement: Supplementary file 1 — Supplementary Material 1 [file 41598_2024_80853_MOESM1_ESM.docx]

**APPENDIX A**

The following Figures A1 And A2, present the comparative analysis of the transient pressure behavior and corresponding axial strain measurements across the five pipe materials (steel, copper, PPr, uPVC, and GRP) at the four specific locations ($Z/L$ = 1, 0.75, 0.45, and 0.25) provides a comprehensive understanding of the water hammer phenomenon in these pipeline systems, at a pump flow rate of 60 L/min and a pressure of 5.5 bar.

At the first location $(Z/L$ = 1), closest to the pump, the pressure transients exhibit distinct patterns for the different pipe materials. The copper pipeline displays the highest peak pressure of 15.5 bar, followed by GRP (14.2 bar), steel (13.3 bar), PPr (13.2 bar), and uPVC (10 bar). This trend can be attributed to the variations in the material properties, such as stiffness and wave speed, which influence the magnitude of the pressure spikes during the WH event.

The differences in the attenuation rates can be explained by the damping characteristics of the pipe materials. Materials with higher damping, such as uPVC, tend to dissipate the pressure wave energy more efficiently, leading to a faster reduction in the peak pressures along the pipeline. Conversely, the stiffer materials, like steel, GRP and copper, exhibit lower damping and, consequently, slower attenuation of the pressure transients.

These findings have important implications for the design and operation of pipeline systems. The selection of the appropriate pipe material can significantly influence the magnitude and distribution of transient pressures, which is crucial for mitigating the risks of water hammer events. The results presented in this study can assist engineers in making informed decisions regarding the selection of pipe materials and the implementation of effective pressure surge protection measures.


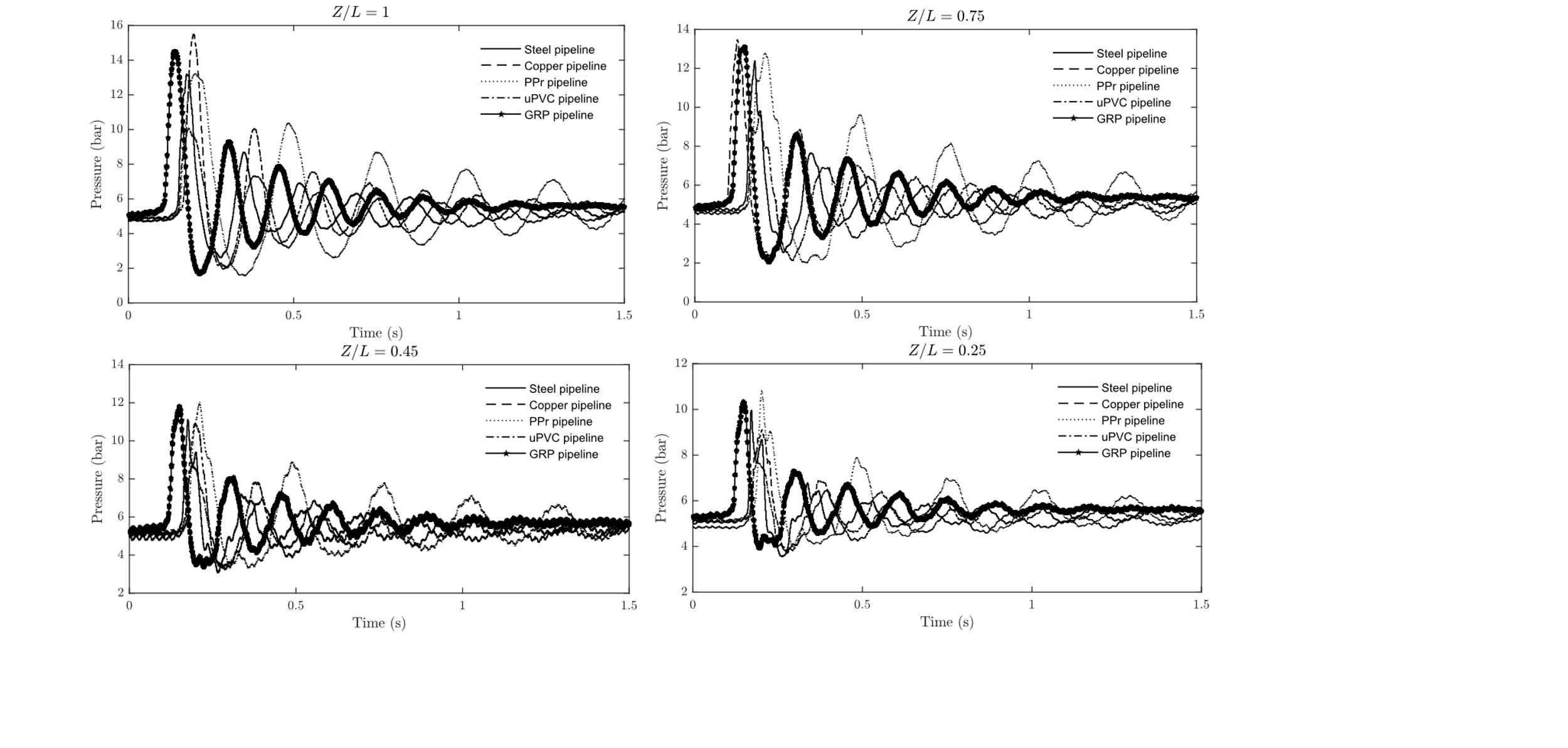


Figure A1: Experimental comparison between the results of transient pressures measured at four specific locations for a pump flow rate of 60 L/min and a pressure of 5.5 bar.


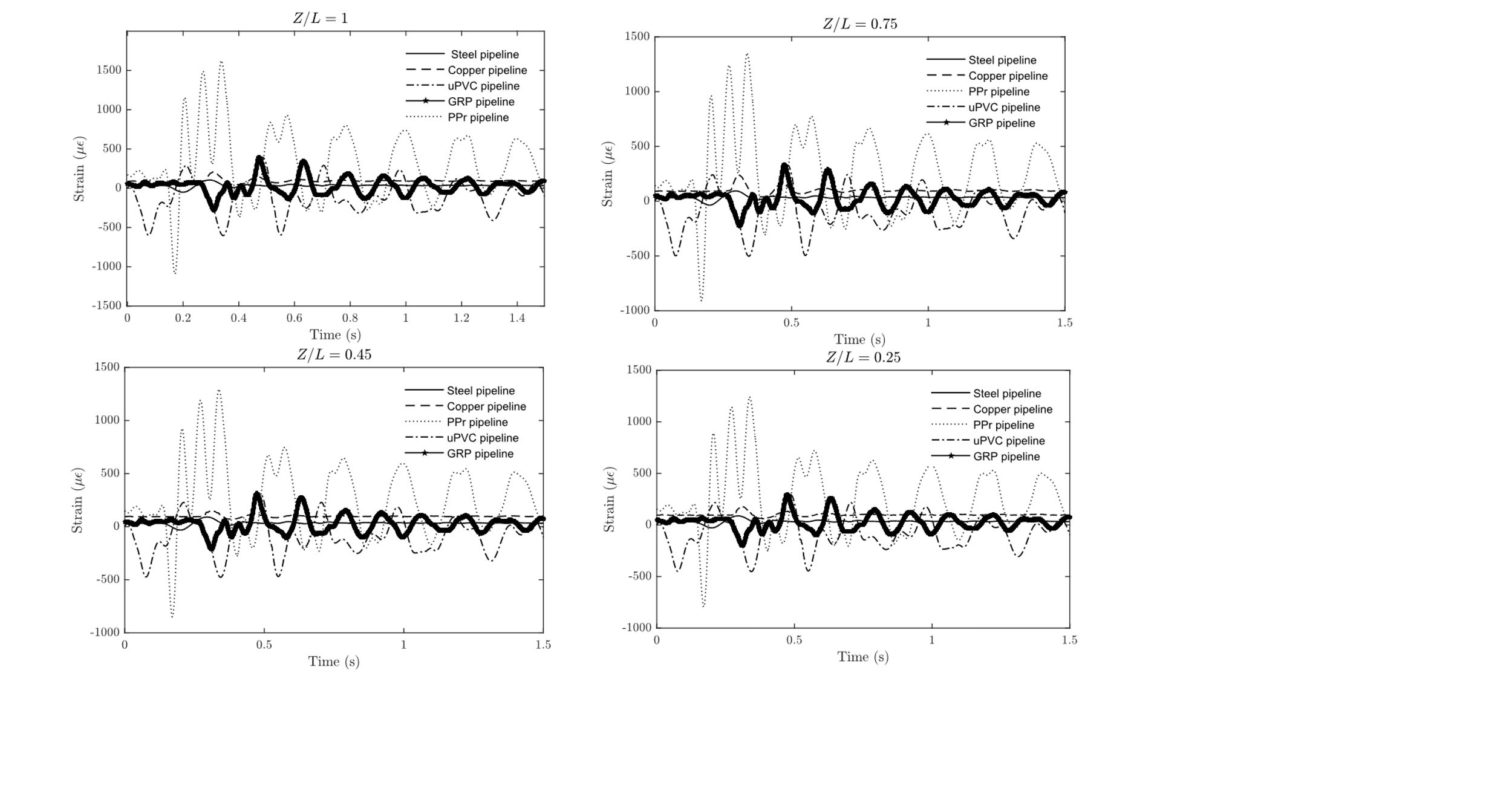


Figure A2: Experimental comparison between the results of corresponding axial strain measurements at four specific locations for a pump flow rate of 60 L/min and a pressure of 5.5 bar.

The following Figures A3 and A4, present the comparative analysis of results for each pipeline materials at the different locations. for transient pressures measured and corresponding axial strain for a pump flow rate of 60 L/min and a pressure of 5.5 bar.

The experimental results obtained for the galvanized steel pipeline under the specified operating conditions of 60 L/min pump flow rate and 5.5 bar pressure provide valuable insights into the transient pressure behavior within the system. By measuring the pressures at four distinct locations along the pipeline, the study was able to capture the spatial and temporal variations in the WH phenomenon.

The pressure transients observed at the four measurement points exhibit distinct patterns, reflecting the complex wave propagation and reflection processes occurring within the pipeline. At location 1, closest to the pump, the pressure spike during the water hammer event is the most pronounced, reaching a peak value of 13 bar. This is consistent with the expected behavior, as the pressure wave originates from this point due to the sudden valve closure.

Moving downstream to locations 2, 3, and 4, the pressure transients show a progressive attenuation in the amplitude of the pressure spikes. At location 2, the peak pressure is reduced to 12.2 bar, while at locations 3 and 4, the peak pressures are further diminished to 11 bar and 10 bar, respectively. This can be attributed to the dissipation of the pressure wave energy due to pipe wall friction and other resistive forces along the pipeline.


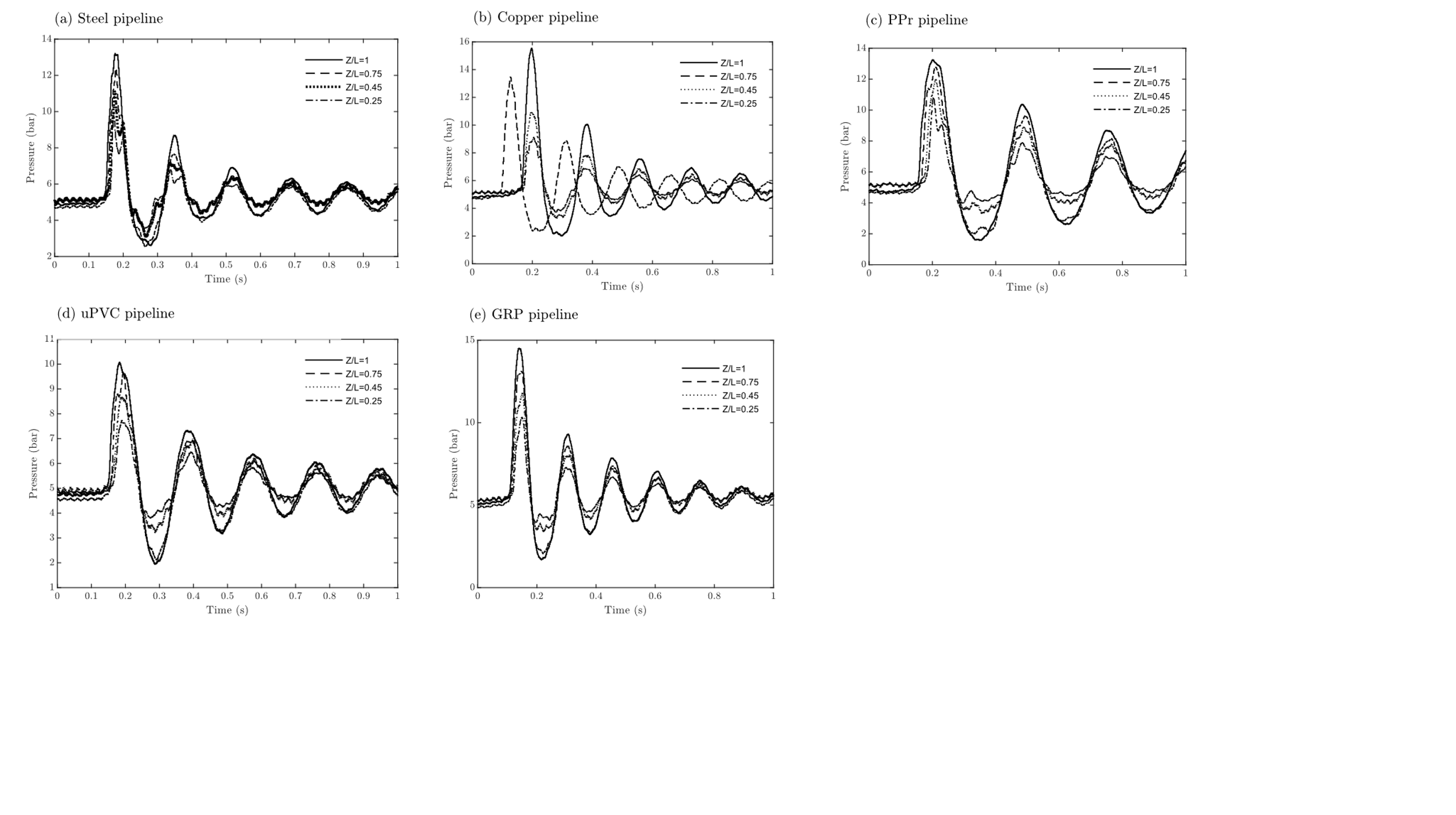


Figure A3: Experimental comparison between the results of transient pressures measured at four specific locations*:* (a) Steel pipeline. (b) Copper pipeline. (c) PPr pipeline. (d) uPVC pipeline. (e) GRP at 60 L/min pump flow rate and a pressure of 5.5 bar.


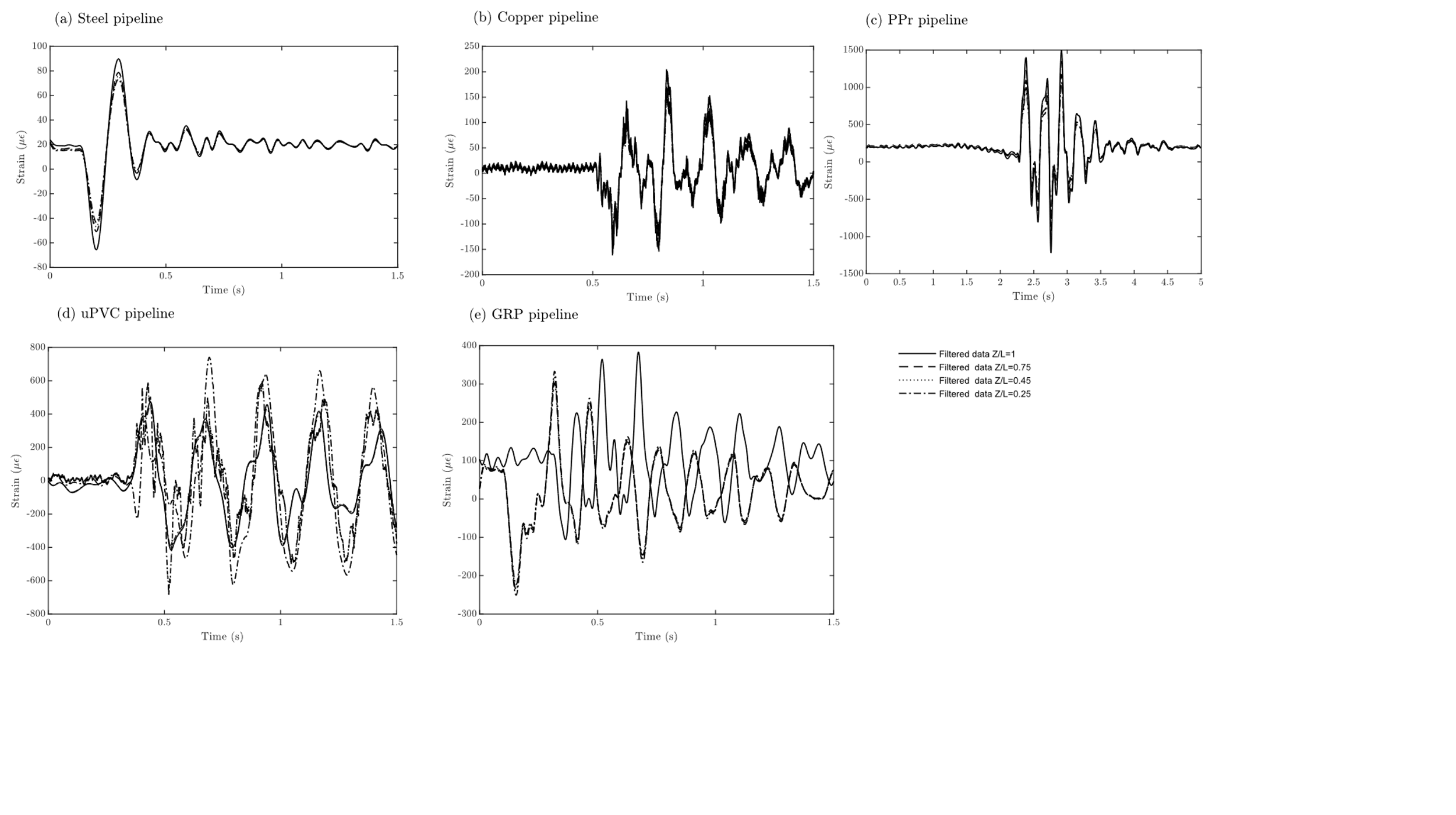


Figure A4: Experimental comparison between the results of strain measured at four specific locations: (a) Steel pipeline. (b) Copper pipeline. (c) PPr pipeline. (d) uPVC pipeline. (e) GRP at 60 L/min pump flow rate and a pressure of 5.5 bar.
